# Ultrahigh‐Throughput Detection of Enzymatic Alcohol Dehydrogenase Activity in Microfluidic Droplets with a Direct Fluorogenic Assay

**DOI:** 10.1002/cbic.202100322

**Published:** 2021-10-13

**Authors:** Miriam Klaus, Paul Jannis Zurek, Tomasz S. Kaminski, Ahir Pushpanath, Katharina Neufeld, Florian Hollfelder

**Affiliations:** ^1^ Department of Biochemistry University of Cambridge 80 Tennis Court Road CB2 1GA Cambridge UK; ^2^ Johnson Matthey Plc 260 Cambridge Science Park CB4 0WE Cambridge UK; ^3^ Current address: Janssen Pharmaceutica Turnhoutseweg 30 2340 Beerse Belgium; ^4^ Current address: ICB Nuvisan GmbH Müllerstraße 178 13353 Berlin Germany; ^5^ Current address: BioNTech Cell & Gene Therapies GmbH An der Goldgrube 12 55131 Mainz Germany; ^6^ Current address: Department of Environmental Microbiology and Biotechnology Institute of Microbiology Faculty of Biology University of Warsaw Miecznikowa 1 02-096 Warsaw Poland

**Keywords:** alcohol dehydrogenase, directed evolution, droplet microfluidics, functional metagenomics, ultrahigh-throughput screening

## Abstract

The exploration of large DNA libraries of metagenomic or synthetic origin is greatly facilitated by ultrahigh‐throughput assays that use monodisperse water‐in‐oil emulsion droplets as sequestered reaction compartments. Millions of samples can be generated and analysed in microfluidic devices at kHz speeds, requiring only micrograms of reagents. The scope of this powerful platform for the discovery of new sequence space is, however, hampered by the limited availability of assay substrates, restricting the functions and reaction types that can be investigated. Here, we broaden the scope of detectable biochemical transformations in droplet microfluidics by introducing the first fluorogenic assay for alcohol dehydrogenases (ADHs) in this format. We have synthesized substrates that release a pyranine fluorophore (8‐hydroxy‐1,3,6‐pyrenetrisulfonic acid, HPTS) when enzymatic turnover occurs. Pyranine is well retained in droplets for >6 weeks (i. e. 14‐times longer than fluorescein), avoiding product leakage and ensuring excellent assay sensitivity. Product concentrations as low as 100 nM were successfully detected, corresponding to less than one turnover per enzyme molecule on average. The potential of our substrate design was demonstrated by efficient recovery of a *bona fide* ADH with an >800‐fold enrichment. The repertoire of droplet screening is enlarged by this sensitive and direct fluorogenic assay to identify dehydrogenases for biocatalytic applications.

## Graphical abstract

**An ultrahigh throughput** screening assay of alcohol dehydrogenases in microfluidic droplets becomes possible with substrates that release a pyranine fluorophore. With a 100 nM detection threshold and no appreciable leaking even after 6 weeks, less than one turnover per enzyme molecule on average is robustly detectable.

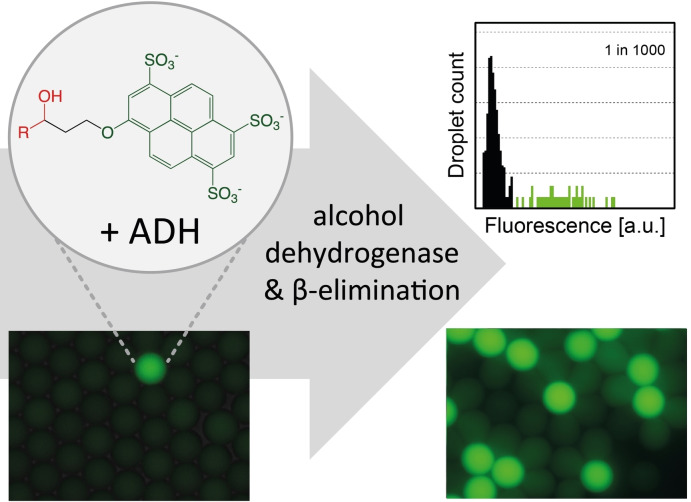

## Introduction

Ultrahigh‐throughput screening of enzymes with droplet microfluidics has established itself as a valuable tool for interrogation of libraries with >10^7^ members. By miniaturizing reaction compartments to pL volumes, droplet microfluidics drastically increases throughput while reducing reagent consumption by 6–8 orders of magnitude.^[1]^ These advances improve the success rate of screening campaigns by enabling the testing of larger sample numbers.^[2]^ Droplet screening has been used for the directed evolution of existing biocatalysts^[3]^ and for the functional identification of novel biocatalysts from metagenomic DNA libraries.^[4]^ The latter holds enormous potential, because the identification of new catalysts enables the functional annotation of previously unknown sequence space,^[5]^ thereby complementing the continuously increasing amount of available – yet often incompletely or incorrectly annotated – sequence data.^[6]^

In order to make droplet microfluidics a universal discovery tool, several challenges remain:

- *Expanding the scope of reactions that can be assayed*. The currently available droplet assays do not cover the many different chemistries encountered in natural repertoires. Most examples focus on hydrolases,^[2a]^ while oxidoreductases have been mainly targeted by taking advantage of coupled reactions[^3a^, ^3c^] or by applying detection modes that are less sensitive than fluorescence and only allow a lower throughput.[^3c^, ^7^] The only other direct fluorogenic assay for oxidoreductases is based on Amplex® UltraRed,^[8]^ the product of which is shown to be depleted quickly from droplets due to leaking.^[9]^
- *Establishing ‘leakage‐free’ assay designs*. The UV/Vis‐active reaction product has to remain contained in the droplet in which it was generated to quantitatively label the library member for selection. Leakage of – typically apolar – products from the aqueous interior of a reactive droplet across the oil phase into that of another droplet removes the identifier of a ‘hit’ and, when such inter‐droplet transfer is rapid, imposes a limit to the ability to discover low activities.
- *Increasing the detection limit*. Assay sensitivity determines whether ‘hits’ can be identified: sensitive detection of initially weak, promiscuous activities toward non‐natural substrates catalysed by heterologously expressed enzymes in directed evolution or functional metagenomics is necessary for successful enrichment. Fluorescence detection is often preferred over colorimetric alternatives due to higher sensitivity,[^3c^, ^3e^, ^10^] with product detection thresholds in the nM range. Combined with small droplet volumes, a very low number of molecules is required for a fluorescence read‐out: just 3000 product molecules suffice.[^4b^, ^11^] With alternative detection modes, the limits are in the low μM range and 100‐ to 1000‐fold larger droplet volumes are essential. This puts much more stringent requirements on enzyme activity and the minimal detectable number of product molecules,[^3c^, ^7^, ^12^] potentially precluding the identification of promiscuous or low initial activities. Finally, slower rates for sorting *via* absorbance (300 Hz),^[3c]^ electrochemistry (10 Hz)^[7]^ or mass‐spectrometry (0.7 Hz)^[12]^ make fluorescence‐based selections with sorting rates well above 1000 Hz attractive.

Here, we expand the range of enzymes that can be sensitively assayed *via* fluorescence in droplets to encompass the biocatalytically important class of alcohol dehydrogenases (ADHs). ADHs are widely used in organic synthesis as biocatalysts for preparation of enantiomerically pure chemicals.^[13]^ We present the design, synthesis and optical characterization of novel pyranine‐based fluorogenic substrates and demonstrate their utility for the ultrahigh‐throughput detection of ADHs by enrichment experiments using droplet microfluidics.

## Results

### Synthesis of fluorogenic ADH substrates

**Figure 1:**
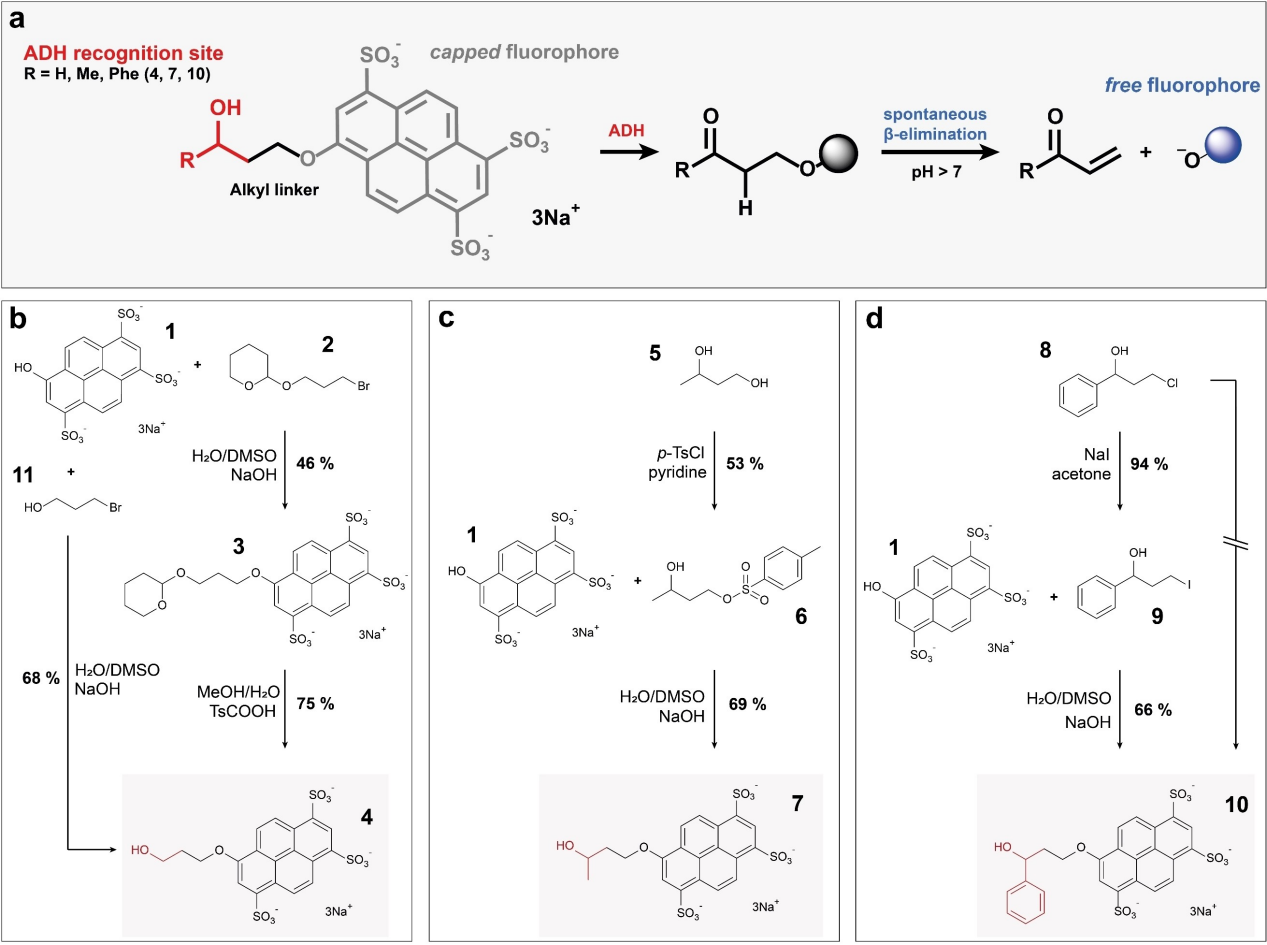
Fluorogenic ADH substrates for droplet‐based screenings. (a) A general substrate structure and assay design is shown. The ADH recognition moiety (red) is covalently attached to pyranine **1** (shown in *grey* for the alkylated non‐fluorescent form, shown in blue for the fluorescent ‘free’ dye) *via* a short alkyl linker (black). Oxidation of the alcohol to a ketone or aldehyde results in instable β‐aryloxy carbonyl products that rapidly undergo β‐elimination under basic conditions (pH>7). (b) Two routes towards H‐HTPS **4** starting from either protected or unprotected 3‐bromo‐1‐propanol **2** or **11**, respectively. (c) Synthesis of Me‐HTPS **7** via activation of 1,3‐butandiol **5** as a tosylate **6**. (d) Synthesis of Phe‐HTPS **10** via activation of chloride precursor **8** as an iodine **9**; shortening the route starting directly from the chloride **8** failed.

We identified pyranine **1** (8‐hydroxy‐1,3,6‐pyrenetrisulfonic acid trisodium salt HPTS)^[14]^ – a non‐toxic fluorophore,^[15]^ a dye familiar from yellow highlighter pens – as a promising candidate for a ‘droplet‐compatible’ fluorophore that could report on reaction progress, if built into a substrate molecule that would allow its release after an enzymatic reaction. Inspired by previously reported fluorogenic ADH assays,^[16]^ we synthesized a set of three ADH substrates for droplet screening. Each of them contains a hydroxy group in the context of an ADH recognition site, covalently attached to pyranine via a short alkyl linker (Figure 1a). In the three designed substrates (see Figure 1b–d), the biocatalytic oxidation of the alcohol moiety to a ketone or aldehyde results in unstable β‐aryloxy carbonyl products that rapidly undergo β‐elimination under basic conditions (pH >7), thereby liberating pyranine which allows the measurement of turnover. The compounds **4**, **7** and **10** differ from one another with respect to the steric demands around the hydroxy group of the ADH recognition site, so that a range of molecular recognition features is covered by the collection: as an example of a primary alcohol substrate H‐HTPS **4** was generated; Me‐HTPS **7** and Phe‐HTPS **10** were produced as examples for secondary alcohols containing either a small methyl (**7**) or a sterically more demanding phenyl group (**10**), respectively. In detail, commercially available THP‐protected 3‐bromo‐1‐propanol **2** was used as a precursor for the synthesis of compound **4**. In case of substrates **7** and **10**, activation of commercially available materials was initially required and achieved either by selective tosylation of the primary hydroxyl group of 1,3‐butandiol (**5**) or chloride halogen exchange in compound **8** giving the respective iodine derivative **9**. Using these precursors, displacement of either bromide, tosylate, or iodine by pyranine in DMSO‐NaOH‐water‐mixtures gave the desired ethers **3**, **7** and **10** in high purity with reasonable yields (44–69 %); subsequent THP‐deprotection of product **3** under acidic conditions gave the targeted substrate **4**. Aiming to shorten the 2‐step synthesis of compound **4**, an alternative 1‐step approach starting from unprotected 3‐bromo‐1‐propanol **11** was successfully demonstrated. An analogous one‐step approach to compound **10** using chloride displacement in precursor **8** was tested but failed to give pure product due to partial alkylation of pyranine. We have found these syntheses to be easily adopted by non‐specialist laboratories without sophisticated synthesis set‐ups: all reactions containing pyranine or its ethers were performed in shaking incubators without the need for any specialised synthetic equipment. Isolation of the desired products was achieved solely by precipitation and washing steps without any column purification. Compared to e. g. the synthetic challenges that arise with derivatisation of fluorescein (based on resonance, solubility and the different reactivities of the two hydroxy groups that need to be derivatised), the presented synthesis not only allows the rapid generation of extended substrate libraries, but can also be carried out by scientists with minimal organic chemistry training.

### Optical readout of ADH‐catalysed turnover

**Figure 2:**
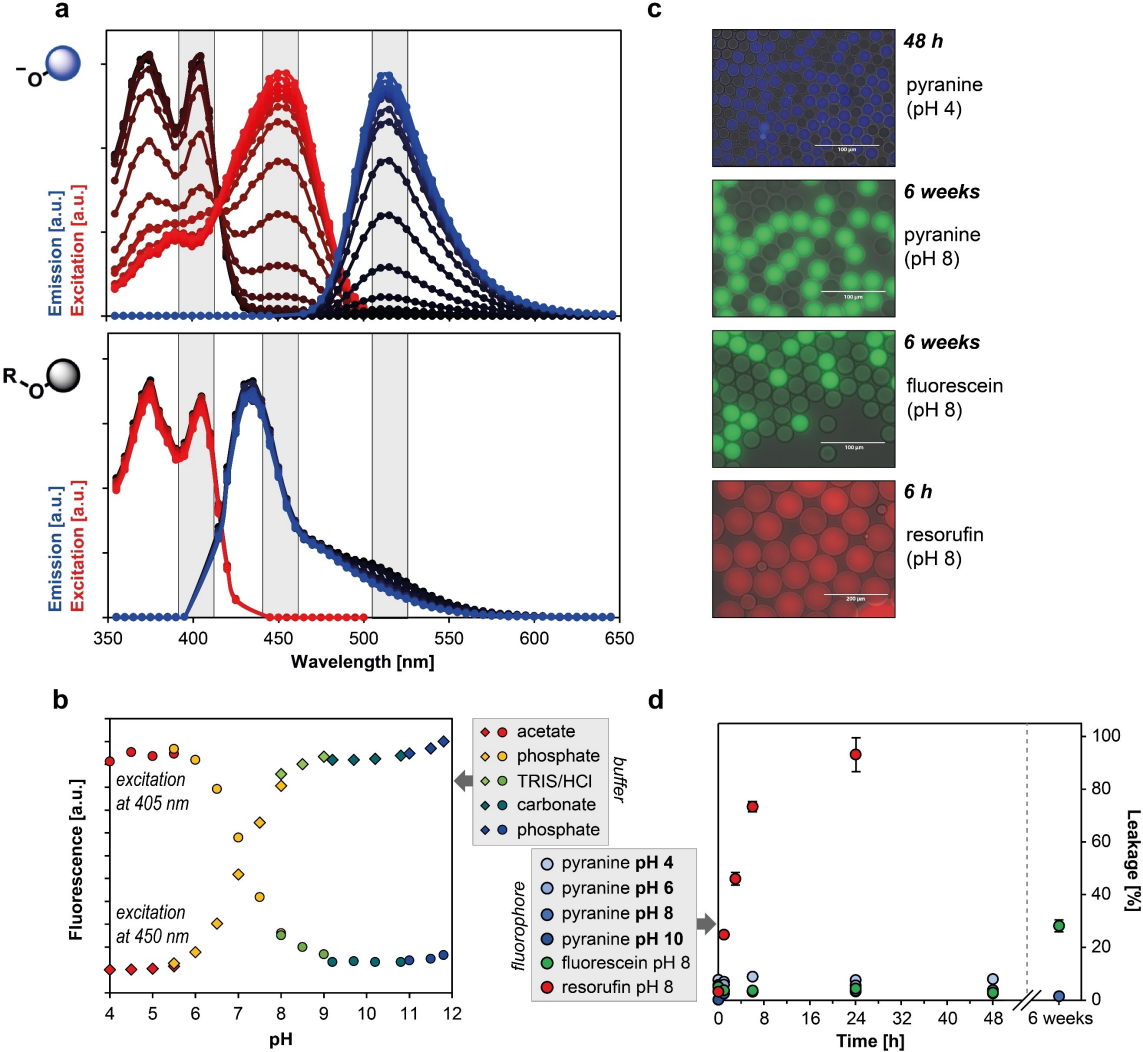
Optical properties of pyranine and substrates. (A) Excitation and emission spectra of pyranine **1** and substrate **7** (serves as a proxy; data representative for all ethers in this study) in different buffers with pH values varying between pH 4.0 and pH 11.8; lower pH values are indicated by increasingly darker graph shade and emission data for pyranine **1** recorded using excitation at 450 nm. (B) Emission values shown as a function of pH at two different excitation wavelengths corresponding to different protonation states of pyranine; buffer type as indicated by colour. (C) Visualization of droplets containing pyranine dissolved in buffer with different pH values confirms ability to detect pyranine over broad pH range; fluorescein and resorufin samples were included as positive controls for leakage experiments at pH 8.0. (D) Leakage of fluorescent dyes from aqueous droplets (n=10, error bars show standard deviation) as a function of time; results indicate stable retention of pyranine **1** in droplets under all pH values tested.

The photophysical properties of pyranine **1** as the final reaction product were investigated and compared to substrates **4**, **7**, and **10**. In case of the ‘free’ dye, a pH independent emission maximum can be observed at 515 nm while the maximum excitation wavelength varies depending on which side of the apparent pyranine p*K*_a_ (of ∼7.3) measurements were taken; for the protonated form the maximum was observed at 405 nm, while the deprotonated form has a maximum at 450 nm (Figure 2a). Alkylation of the 8‐hydroxy group of pyranine results in an excitation maximum at 405 nm and a hypsochromically shifted emission maximum at 435 nm compared to the parent pyranine due to loss of conjugation with the 8‐hydroxyl group (Figure 2). Pyranine **1** thus differs in its optical properties (summarised in Table 1) to the widely used phenolic dyes, in that for the latter only the de‐protonated anionic forms show fluorescence, whereas pyranine fluorescence is observed regardless of its protonation state. This unique optical feature enables the detection of pyranine over a broad pH range (Figure 2b) resulting in its numerous applications as a pH indicator in biological systems^[17]^ and for measuring enzymatic activities across a range of pH conditions, including the acidic range.

**Table 1:** Optical properties of substrates **4**, **7** and **10** compared to the reaction product **1**.

Compound	λ_ex_ [nm]	λ_em_ [nm]	Stokes shift [nm]
HTPS **1**	405/450	515	110/60
HTPS ethers **4**, **7** and **10**	405	435	30

### Long‐term retention of pyranine in droplets

The escape of product from droplet compartments can endanger the success of screening experiments because inter‐droplet exchange of the optically active product renders all droplets identical, and ‘hits’ cannot be identified. For example, fluorescein is retained sufficiently on timescales of a few days,[^4b^, ^9a^] whereas resorufin leakage can already be detected after a few hours in slightly basic conditions. To investigate the leaking properties of pyranine (**1**) at different pH values, the compound was encapsulated into microfluidic droplets and the resulting droplet population mixed with a second droplet population not containing the dye. The rate of fluorophore exchange between the two co‐incubated droplet populations was monitored at pH 4.0, 6.0, 8.0, and 10.0 with a fluorescence microscope. The percentage of leakage was quantified by measuring the average grey values of 10 droplets from each population and calculating the relative differences in fluorescence intensity between these corrected by background intensity. After 48 h of incubation, no detectable exchange occurred between droplets at any of the tested pH values. Similarly, a fluorescein control at pH 8.0 showed no exchange between droplets during the 48 h time frame, while resorufin ended up equally distributed between both droplet populations after just 24 h (Figure 2c–d). After 6 weeks, 28 % leakage was detected for fluorescein (Figure S1), while droplets filled with pyranine **1** showed less than 4 % leakage under the same conditions (Figure S1). The experiment was repeated with substrate **7** at pH 8.0 and revealed only 6 % leakage after 6 weeks of incubation, indicating that not only product pyranine (parent) is perfectly retained in the droplets, but also the respective substrate ethers **4**, **7** and **10**. This is the longest retention of any fluorophore achieved in droplets to date and an order of magnitude longer compared to fluorescein that is currently the analyte of choice for monitoring slow reactions. The maintenance of product compartmentalisation over such long time periods enables more sensitive long‐term assays over a larger pH range, e. g. to evaluate weak promiscuous enzymatic activities or poorly expressed biocatalysts.

### Detection limit, dynamic range and enrichment

**Figure 3:**
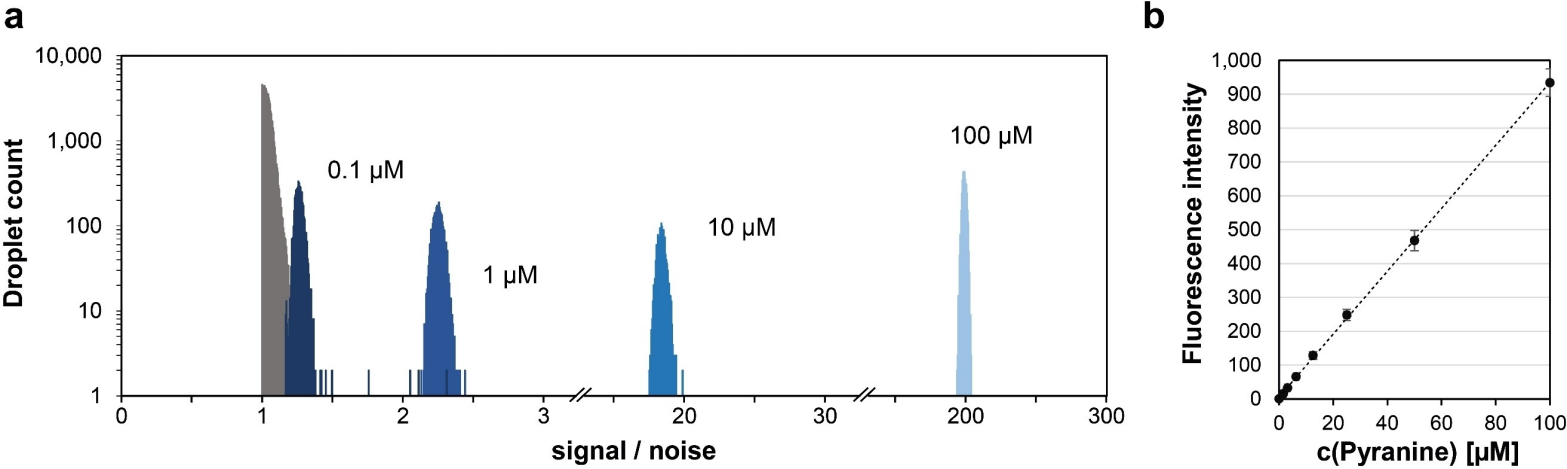
Detection limits and dynamic range of pyranine in droplets. (a) Signal to noise ratios of separately generated droplet populations containing different pyranine concentrations; signal intensities are presented relative to instrument baseline (normalized to 1, shown in grey) and measurements were performed using a 488 nm laser for excitation. (b) A pyranine calibration curve obtained from microwell plate measurements; pyranine fluorescence as a function of pyranine concentration is linear up to 100 μM dye (λ_ex_ 450 nm/λ_em_ 515 nm). Measurements were taken in two independent replicates, error bars represent the standard deviation.

The minimal product concentration required to identify droplets with active enzyme was determined by measuring the fluorescence values of several droplet populations containing different pyranine concentrations. An overlay of these distribution functions is shown in Figure 3; signal intensities were adjusted relative to a normalized instrument baseline set to 1. In case of 0.1 μM pyranine, less than 20 % overlap was observed between the droplet population signal set and baseline variation. This minimal detectable concentration corresponds to 1.8×10^5^ pyranine molecules in a 3 pL droplet. To maintain droplet monoclonality in our droplet screening set‐up, a single cell must provide a sufficient amount of enzyme to enable a reaction read‐out. Here, if intracellular recombinant expression levels of ∼10^5^–10^6^ molecules per cell can be assumed,^[3c]^ less than one substrate turnover per enzyme molecule would release sufficient pyranine to exceed the threshold to be detected. A calibration curve using microtiter plate measurements confirms that pyranine fluorescence increases proportionally with the pyranine concentration up to a dye concentration of 100 μM (Figure 3b). Our data demonstrate sub‐μM sensitivity and a wide, quantitative dynamic range over three orders of magnitude suggest that a wide range of activity levels can be reliably detected.

**Figure 4:**
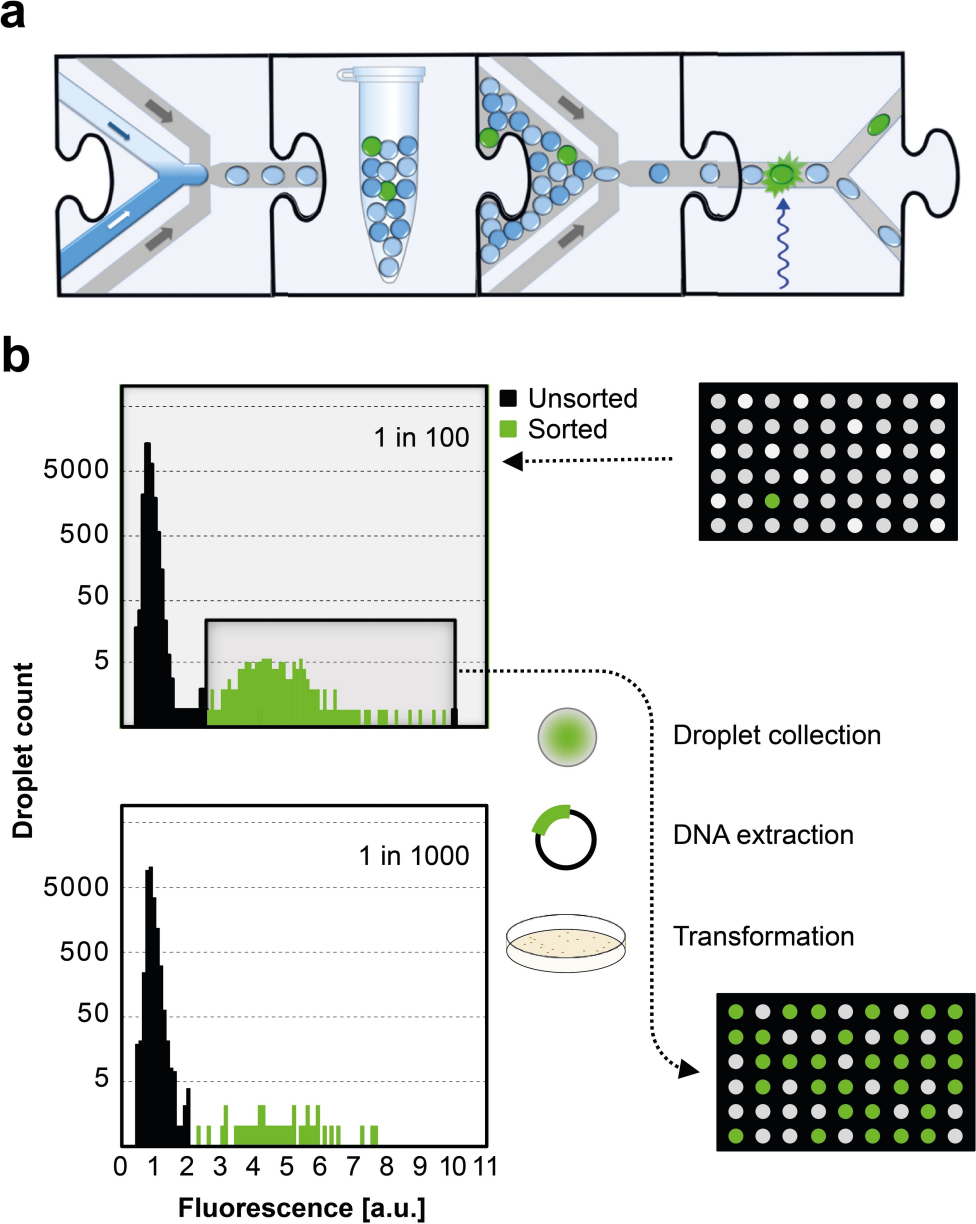
Enrichment of active enzyme in a model selection. (a) Microfluidic workflow represented with jigsaw pieces each corresponding to a microfluidic step.^[1a]^ First, single cells are co‐encapsulated with substrate and lysis solution that breaks down the cell wall and allows encounter of enzyme and substrate.^[19b]^ Next, droplets are incubated in bulk to allow for reaction progress. Following incubation, the re‐injection of droplets into a sorting chip enables selection of highly fluorescent droplets; i. e. droplets containing active enzyme. The actual chip designs are shown in the supplementary Figure S2. (b) Two reference libraries containing a 1 : 100 and a 1 : 1000 mixture of cells expressing or lacking an ADH were prepared and used for droplet generation aiming for single cell occupancy together with lysis agent and reaction mix; substrate **4** was used for the lower and substrate **7** for the higher dilution. Reactions were allowed to proceed for 48 h, resulting in the development of a detectable fluorescent signal in the ADH containing droplets. Samples were subjected to sorting and subsequent plasmid recovery, leading to a significant enrichment: 90 % and 47 % of obtained hits contained the ADH gene compared to the initial 1 % and 0.1 % content of the respective starting points. Microtiter plate visualization exemplarily illustrates the composition of cultures before and after sorting for the 1 : 100 dilution experiment.

To validate the application of the new substrates, a model selection was carried out. An *E. coli* culture expressing a genuine ADH (UniProt B5ICK5, in the vector pASK‐IBA63b+) was used as a positive control. Cells bearing an expression plasmid were mixed with cells containing the same pASK vector without an insert (as a negative control) in ratios 1 : 100 and 1 : 1000. Using these samples, droplets containing single cells along with lysis agent and reaction mix were generated using the device shown (Figure 4a, Figure S2), with substrates **4** and **7** in two different dilutions. Samples were incubated for 48 h at room temperature to allow the liberation of biocatalysts from cells upon lysis and for the catalytic alcohol oxidation to take place, resulting in a detectable fluorescent signal in the ADH‐containing droplets. On‐chip sorting with fluorescence‐activated droplet sorting (FADS) (Figure 4a, Figure S2) was followed by plasmid recovery and transformation of the recovered plasmids into *E. coli*. Significant enrichments were observed: 90 % (79 out of 88 colonies) and 47 % (15 out of 32 colonies) of the recovered hits contained the ADH gene compared to the initial 1 % and 0.1 % content of the respective starting dilutions (Figure 4b; Tables S6 and S7). These numbers correspond to a 869‐ and 882‐fold enrichment (calculated according to Baret *et al*.^[10]^) or 90‐fold and 469‐fold using the formula of Zinchenko *et al*.^[18]^ The enrichment ratios achieved are comparable to previous droplet selections[^3c^, ^19^] that have been successfully carried forward to library screenings and suggest low false positive rates in the microfluidic set‐up. We conclude that pyranine‐based substrates are suitable to detect rare events of biocatalytic ADH activity in libraries.

### Discussion

The establishment of the first single‐step fluorogenic assay for ADHs amenable to ultrahigh throughput in droplets will provide ready access to highly desirable biocatalysts for the dehydrogenation of alcohols and the reverse reaction, ketoreduction. Broadening the scope of droplet microfluidics beyond the most frequently studied enzymatic transformations^[2a]^ to hitherto unavailable oxidoreductases will open avenues for the discovery of new enzymes for biocatalysis. Despite the importance of ADHs in biocatalysis for synthetic chemistry,[^13b^, ^13c^] combinatorial exploration of libraries has thus far either relied on *in vivo* selections (based on growth advantages through release of a carbon source by the enzymatic reaction^[20]^) or relatively low throughput assays using colorimetric detection on agar or microtiter plates.^[20b]^ As the chances of finding ADHs in metagenomic samples are likely to be low and high throughput assays are as yet unavailable,^[21]^ sequence‐based approaches^[22]^ have naturally dominated enzyme discovery over functional metagenomic strategies in the past, despite promising examples for the latter at ultrahigh throughput.^[4b]^ In this work, we have demonstrated enzyme screens suitable for discovery of novel ADHs in functional metagenomics or in directed evolution^[23]^ experiments. Substrates **4**, **7** and **10** lead to a fluorescent pyranine product that remains encapsulated in droplets for a long time. The retention time of pyranine in droplets is the longest measured thus far, ∼14‐fold longer than fluorescein, the dye known to be least leaky in emulsion‐compartmentalised assays.[^3d^, ^4b^, ^19b^, ^24^] Other fluorogenic leaving groups, namely fluorescein or coumarin derivatives (such as 7‐hydroxycoumarin‐3‐carboxylate^[3d]^ and 3‐amino‐7‐hydroxycoumarin^[27]^) showed leakage on timescales between minutes and days or required synthetic efforts (e. g. 7‐aminocoumarin‐4‐methansulfonate,^[25]^ 6,8‐difluoro‐7‐hydroxycoumarin‐4‐methansulfonate^[26]^). Beyond ADHs, replacing previously used fluorophores with pyranine could extend the time windows for slow reactions: broadly from hours (coumarins) to days (fluorescein) to weeks (pyranine). The ADH used in this study reacted with the fluorogenic pyranine substrates and also turns over substrates that do not carry the fluorophore. The same observation has been made for ADH selections from metagenomic libraries,^[28]^ suggesting that the use of the substrates **4**, **7** and **10** does not elicit enzymes that are exclusively turning over substrates containing a pyranine fluorophore, but catalysts that are able to oxidise a range of alcohol substrates.

The optical properties of pyranine recommend it as a convenient assay choice: the large Stokes shift of pyranine makes it less conducive to self‐quenching^[29]^ and its longwave excitation and emission maxima ensure negligible interference due to low spectral overlap with biological materials.^[30]^ The linearity of the pyranine product signal means that quantitative thresholds can be reliably set, and activity differences can be precisely measured. As pyranine fluoresces in both acidic and alkaline pH on either side of its p*K*_a_ (∼7.3, Figure 2a/b),^[29]^ enzymatic activities can be screened across a wide pH range, with the caveat that the spontaneous β‐elimination requires base catalysis and its rate will drop under more acidic conditions. Thus, the rate limiting step at neutral or low pH may not be the enzymatic reaction. However, in workflows where the reaction is terminated by quenching with acid, reaction product can still be seen at low pH.

The sensitivity of pyranine‐based substrates can be compared to alternative assays that monitor redox reactions of ketone groups and are currently used in droplet screening. The detection limit of 0.1 μM is 100‐fold more sensitive than in a coupled absorbance assay (10 μM),[^3c^, ^3e^, ^31^] 10‐fold compared to an electrochemical assay (1 μM)^[7]^ and 300‐fold compared to a mass spectrometric set‐up (30 μM).^[12]^ Since the droplet sizes in these three formats vary, comparisons are even more pronounced when the volumes are taken into account: 2×10^5^ molecules are needed to detect pyranine (in 3 pL droplets), but 10^9^ molecules are needed in AADS (in 180 pL),[^3c^, ^3e^] 2×10^10^ in electrochemical detection (in 30 nL)^[7]^ and 5×10^11^ in mass spectrometry (in 25 nL).^[12]^ Less than one molecule of pyranine substrate has to be turned over per enzyme to achieve detection, while for other detection modes 1300 (for AADS),[^3c^, ^3e^] 22,600 (electrochemically)^[7]^ and 565,000 (by mass spectrometry)^[12]^ substrate molecules have to be converted by each enzyme molecule (see Table S1, SI for a summary). Thus, pyranine is the most sensitive detector of redox reactions of ketone groups available for droplet screening, where sorting rates above kHz can be realised.

Our straightforward and versatile synthesis gives access to new substrates for sensitive ADH screening in droplets, where metagenomic or directed evolution libraries will be interrogated at ultrahigh throughput. The synthesis can be quickly adapted to incorporate further structural variation around the carbonyl group (as demonstrated with the family of substrates **4**, **7**, and **10**), so that a variety of molecular recognition features matching the eventual target application become accessible synthetically. It will be interesting to see which specificities of ADH enzymes will be elicited when collections of substrates (all leading to the same pyranine product) are used as a bait in enzyme discovery campaigns. For now, an additional reaction type has become amenable to droplet screening in microfluidics and screening campaigns of ADHs will be able to take advantage of screening large libraries (>10^7^) compartmentalised in picoliter volumes at ultrahigh frequencies (>kHz).

## Experimental Section

**Substrate synthesis**: In a procedure based on reference [32], pyranine (1.0 eq.) was dissolved in DMSO to a concentration of 30 g/L by vigorous mixing over two hours. Subsequently an aqueous NaOH solution (50 % w/w, 1.8 eq. of base) was added and stirring was continued for another 30 min before the respective coupling partner (bromides **2** or **11**, tosylate **6** or iodine **9**, 2.0 eq.) was added in one portion and the reaction was allowed to proceed for two days at 24 °C until pyranine fluorescence had disappeared. Acetone (5 reaction volumes) was added to the reaction mixture to precipitate the product. The solid product was then recovered via centrifugation (4000 rpm, RT, 5 min) and removal of the supernatant. The precipitate was re‐dissolved in a minimal amount of water and the aforementioned precipitation procedure repeated with acetone (albeit now with 2.5 reaction volumes). The pellet thus obtained was washed twice with acetone (1.25 reaction volumes) *via* repeated sequences of vortexing, centrifugation and removal of the supernatant. Finally, the product was dissolved in water (0.5 reaction volumes) and freeze‐dried to give the desired products **4**, **7** and **10**, respectively, with 40–70 % overall yields as yellow‐orange powders. Detailed procedures and analytical characterisation can be found in the SI.

**Microfluidic droplet assays**: Briefly, chip devices were made by soft lithography from poly(dimethyl)siloxane (PDMS) based on designs created with AutoCAD 2018 or DraftSight (Dassault Systems), shown in Figure S2 (SI). The corresponding CAD files can be downloaded from http://openwetware.org/wiki/DropBase. Detailed procedures for chip manufacture, the operation of droplet assays, fluorophore retention assays, the set‐up of catalytic ADH assays (including DNA recovery) and the optical properties of pyranine can be found in the Supporting Information.

## Conflict of interest

The authors declare no conflict of interest.

## Supporting information

As a service to our authors and readers, this journal provides supporting information supplied by the authors. Such materials are peer reviewed and may be re‐organized for online delivery, but are not copy‐edited or typeset. Technical support issues arising from supporting information (other than missing files) should be addressed to the authors.

Supporting Information

## References

[1a] B. Kintses , L. D. van Vliet , S. R. Devenish , F. Hollfelder , Curr. Opin. Chem. Biol. 2010, 14, 548–555; 20869904 10.1016/j.cbpa.2010.08.013

[1b] Y. Schaerli , F. Hollfelder , Mol. BioSyst. 2009, 5, 1392–1404. 20023716 10.1039/b907578j

[2a] S. Neun , P. J. Zurek , T. S. Kaminski , F. Hollfelder , Methods Enzymol. 2020, 643, 317–343; 32896286 10.1016/bs.mie.2020.06.002

[2b] P. Y. Colin , A. Zinchenko , F. Hollfelder , Curr. Opin. Struct. Biol. 2015, 33, 42–51; 26311177 10.1016/j.sbi.2015.06.001

[2c] U. Markel , K. D. Essani , V. Besirlioglu , J. Schiffels , W. R. Streit , U. Schwaneberg , Chem. Soc. Rev. 2020, 49, 233–262; 31815263 10.1039/c8cs00981c

[2d] A. Currin , N. Swainston , P. J. Day , D. B. Kell , Chem. Soc. Rev. 2015, 44, 1172–1239. 25503938 10.1039/c4cs00351a PMC4349129

[3a] A. Debon , M. Pott , R. Obexer , A. P. Green , L. Friedrich , A. D. Griffiths , D. Hilvert , Nat. Catal. 2019, 2, 740–747;

[3b] T. Beneyton , I. P. Wijaya , P. Postros , M. Najah , P. Leblond , A. Couvent , E. Mayot , A. D. Griffiths , A. Drevelle , Sci. Rep. 2016, 6, 27223; 27270141 10.1038/srep27223 PMC4895158

[3c] F. Gielen , R. Hours , S. Emond , M. Fischlechner , U. Schell , F. Hollfelder , Proc. Natl. Acad. Sci. USA 2016, 113, E7383-E7389; 27821774 10.1073/pnas.1606927113 PMC5127370

[3d] F. Ma , M. T. Chung , Y. Yao , R. Nidetz , L. M. Lee , A. P. Liu , Y. Feng , K. Kurabayashi , G. Y. Yang , Nat. Commun. 2018, 9, 1030; 29531246 10.1038/s41467-018-03492-6 PMC5847605

[3e] P. J. Zurek , P. Knyphausen , K. Neufeld , A. Pushpanath , F. Hollfelder , Nat. Commun. 2020, 11, 6023. 33243970 10.1038/s41467-020-19687-9 PMC7691348

[4a] A. S. Tauzin , M. R. Pereira , L. D. Van Vliet , P. Y. Colin , E. Laville , J. Esque , S. Laguerre , B. Henrissat , N. Terrapon , V. Lombard , M. Leclerc , J. Dore , F. Hollfelder , G. Potocki-Veronese , Microbiome 2020, 8, 141; 33004077 10.1186/s40168-020-00911-z PMC7531118

[4b] P. Y. Colin , B. Kintses , F. Gielen , C. M. Miton , G. Fischer , M. F. Mohamed , M. Hyvonen , D. P. Morgavi , D. B. Janssen , F. Hollfelder , Nat. Commun. 2015, 6, 10008. 26639611 10.1038/ncomms10008 PMC4686663

[5] F. Berini , C. Casciello , G. L. Marcone , F. Marinelli , FEMS Microbiol. Lett. 2017, 364. 10.1093/femsle/fnx211 29029060

[6a] N. Zhou , Y. Jiang , T. R. Bergquist , A. J. Lee , B. Z. Kacsoh , A. W. Crocker , K. A. Lewis , G. Georghiou , H. N. Nguyen , M. N. Hamid , L. Davis , T. Dogan , V. Atalay , A. S. Rifaioglu , A. Dalkiran , R. Cetin Atalay , C. Zhang , R. L. Hurto , P. L. Freddolino , Y. Zhang , P. Bhat , F. Supek , J. M. Fernandez , B. Gemovic , V. R. Perovic , R. S. Davidovic , N. Sumonja , N. Veljkovic , E. Asgari , M. R. K. Mofrad , G. Profiti , C. Savojardo , P. L. Martelli , R. Casadio , F. Boecker , H. Schoof , I. Kahanda , N. Thurlby , A. C. McHardy , A. Renaux , R. Saidi , J. Gough , A. A. Freitas , M. Antczak , F. Fabris , M. N. Wass , J. Hou , J. Cheng , Z. Wang , A. E. Romero , A. Paccanaro , H. Yang , T. Goldberg , C. Zhao , L. Holm , P. Toronen , A. J. Medlar , E. Zosa , I. Borukhov , I. Novikov , A. Wilkins , O. Lichtarge , P. H. Chi , W. C. Tseng , M. Linial , P. W. Rose , C. Dessimoz , V. Vidulin , S. Dzeroski , I. Sillitoe , S. Das , J. G. Lees , D. T. Jones , C. Wan , D. Cozzetto , R. Fa , M. Torres , A. Warwick Vesztrocy , J. M. Rodriguez , M. L. Tress , M. Frasca , M. Notaro , G. Grossi , A. Petrini , M. Re , G. Valentini , M. Mesiti , D. B. Roche , J. Reeb , D. W. Ritchie , S. Aridhi , S. Z. Alborzi , M. D. Devignes , D. C. E. Koo , R. Bonneau , V. Gligorijevic , M. Barot , H. Fang , S. Toppo , E. Lavezzo , et al., Genome Biol. 2019, 20, 244; 31744546 10.1186/s13059-019-1835-8 PMC6864930

[6b] A. M. Schnoes , S. D. Brown , I. Dodevski , P. C. Babbitt , PLoS Comput. Biol. 2009, 5, e1000605; 20011109 10.1371/journal.pcbi.1000605 PMC2781113

[6c] K. Bastard , A. A. Smith , C. Vergne-Vaxelaire , A. Perret , A. Zaparucha , R. De Melo-Minardi , A. Mariage , M. Boutard , A. Debard , C. Lechaplais , C. Pelle , V. Pellouin , N. Perchat , J. L. Petit , A. Kreimeyer , C. Medigue , J. Weissenbach , F. Artiguenave , V. De Berardinis , D. Vallenet , M. Salanoubat , Nat. Chem. Biol. 2014, 10, 42–49. 24240508 10.1038/nchembio.1387

[7] H. Goto , Y. Kanai , A. Yotsui , S. Shimokihara , S. Shitara , R. Oyobiki , K. Fujiwara , T. Watanabe , Y. Einaga , Y. Matsumoto , N. Miki , N. Doi , Lab Chip 2020, 20, 852–861. 31984406 10.1039/c9lc01263j

[8] T. Beneyton , F. Coldren , J. C. Baret , A. D. Griffiths , V. Taly , Analyst 2014, 139, 3314–3323. 24733162 10.1039/c4an00228h

[9a] F. Courtois , L. F. Olguin , G. Whyte , A. B. Theberge , W. T. Huck , F. Hollfelder , C. Abell , Anal. Chem. 2009, 81, 3008–3016; 19284775 10.1021/ac802658n

[9b] Y. Skhiri , P. Gruner , B. Semin , Q. Brosseau , D. Pekin , L. Mazutis , V. Goust , F. Kleinschmidt , A. El Harrak , J. B. Hutchison , E. Mayot , J.-F. Bartolo , A. D. Griffiths , V. Taly , J. C. Baret , Soft Matter 2012, 41, 10618–10627;

[9c] P. Gruner , B. Riechers , B. Semin , J. Lim , A. Johnston , K. Short , J. C. Baret , Nat. Commun. 2016, 7, 10392. 26797564 10.1038/ncomms10392 PMC4735829

[10] J. C. Baret , O. J. Miller , V. Taly , M. Ryckelynck , A. El-Harrak , L. Frenz , C. Rick , M. L. Samuels , J. B. Hutchison , J. J. Agresti , D. R. Link , D. A. Weitz , A. D. Griffiths , Lab Chip 2009, 9, 1850–1858. 19532959 10.1039/b902504a

[11] J. J. Agresti , E. Antipov , A. R. Abate , K. Ahn , A. C. Rowat , J. C. Baret , M. Marquez , A. M. Klibanov , A. D. Griffiths , D. A. Weitz , Proc. Natl. Acad. Sci. USA 2010, 107, 4004–4009. 20142500 10.1073/pnas.0910781107 PMC2840095

[12] D. A. Holland-Moritz , M. K. Wismer , B. F. Mann , I. Farasat , P. Devine , E. D. Guetschow , I. Mangion , C. J. Welch , J. C. Moore , S. Sun , R. T. Kennedy , Angew. Chem. Int. Ed. 2020, 59, 4470–4477; 10.1002/anie.201913203 31868984

[13a] Y. G. Zheng , H. H. Yin , D. F. Yu , X. Chen , X. L. Tang , X. J. Zhang , Y. P. Xue , Y. J. Wang , Z. Q. Liu , Appl. Microbiol. Biotechnol. 2017, 101, 987–1001; 28074225 10.1007/s00253-016-8083-6

[13b] B. Wiltschi , T. Cernava , A. Dennig , M. Galindo Casas , M. Geier , S. Gruber , M. Haberbauer , P. Heidinger , E. Herrero Acero , R. Kratzer , C. Luley-Goedl , C. A. Muller , J. Pitzer , D. Ribitsch , M. Sauer , K. Schmolzer , W. Schnitzhofer , C. W. Sensen , J. Soh , K. Steiner , C. K. Winkler , M. Winkler , T. Wriessnegger , Biotechnol. Adv. 2020, 40, 107520; 31981600 10.1016/j.biotechadv.2020.107520

[13c] S. Wu , R. Snajdrova , J. C. Moore , K. Baldenius , U. T. Bornscheuer , Angew. Chem. Int. Ed. 2021, 60, 88–119; 10.1002/anie.202006648 PMC7818486 32558088

[14a] L. D. Lavis , T. J. Rutkoski , R. T. Raines , Anal. Chem. 2007, 79, 6775–6782; 17672523 10.1021/ac070907g PMC2868592

[14b] M. S. Goncalves , Chem. Rev. 2009, 109, 190–212; 19105748 10.1021/cr0783840

[14c] M. V. Sednev , V. N. Belov , S. W. Hell , Methods Appl. Fluoresc. 2015, 3, 042004; 29148519 10.1088/2050-6120/3/4/042004

[14d] T. Terai , T. Nagano , Pfluegers Arch. 2013, 465, 347–359; 23412659 10.1007/s00424-013-1234-z

[14e] B. N. Giepmans , S. R. Adams , M. H. Ellisman , R. Y. Tsien , Science 2006, 312, 217–224; 16614209 10.1126/science.1124618

[14f] J. Jose , K. Burgess , J. Org. Chem. 2006, 71, 7835–7839; 16995693 10.1021/jo061369v

[14g] X. Li , X. Gao , W. Shi , H. Ma , Chem. Rev. 2014, 114, 590–659; 24024656 10.1021/cr300508p

[14h] B. Finkler , C. Spies , M. Vester , F. Walte , K. Omlor , I. Riemann , M. Zimmer , F. Stracke , M. Gerhards , G. Jung , Photochem. Photobiol. Sci. 2014, 13, 548–562; 24469857 10.1039/c3pp50404b

[14i] H. Zhu , R C. Derksen , C. R. Krause , R. D. Fox , R. D Brazee , H. E. Ozkan , J. ASTM Int. 2005, 21 (3), 325–329.

[15a] G. A. Lutty , Toxicol. Appl. Pharmacol. 1978, 44, 225–249; 79242 10.1016/0041-008x(78)90185-0

[15b] H. Behrens , U. Beims , H. Dieter , G. Dietze , T. Eikmann , T. Grummt , H. Hanisch , H. Henseling , W. Käß , H. Kerndorff , C. Leibundgut , U. Müller-Wegener , I. Rönnefahrt , B. Scharenberg , R. Schleyer , W. Schloz , F. Tilkes Hydrogeology J. 2001, 9, 321–325;

[15c] M. S. Field , R. G. Wilhelm , J. F. Quinlan , T. J. Aley , Environ. Monit. Assess. 1995, 38, 75–96. 24197914 10.1007/BF00547128

[16] G. Klein , J. L. Reymond , Bioorg. Med. Chem. Lett. 1998, 8, 1113–1116. 9871718 10.1016/s0960-894x(98)00165-6

[17] J. Han , K. Burgess , Chem. Rev. 2010, 110, 2709–2728. 19831417 10.1021/cr900249z

[18] A. Zinchenko , S. R. Devenish , B. Kintses , P. Y. Colin , M. Fischlechner , F. Hollfelder , Anal. Chem. 2014, 86, 2526–2533. 24517505 10.1021/ac403585p PMC3952496

[19a] J. M. Holstein , C. Gylstorff , F. Hollfelder , ACS Synth. Biol. 2021, 10, 252–257; 33502841 10.1021/acssynbio.0c00538 PMC7901014

[19b] B. Kintses , C. Hein , M. F. Mohamed , M. Fischlechner , F. Courtois , C. Laine , F. Hollfelder , Chem. Biol. 2012, 19, 1001–1009. 22921067 10.1016/j.chembiol.2012.06.009

[20a] M. Wexler , P. L. Bond , D. J. Richardson , A. W. Johnston , Environ. Microbiol. 2005, 7, 1917–1926; 16309390 10.1111/j.1462-2920.2005.00854.x

[20b] A. Knietsch , T. Waschkowitz , S. Bowien , A. Henne , R. Daniel , Appl. Environ. Microbiol. 2003, 69, 1408–1416; 12620823 10.1128/AEM.69.3.1408-1416.2003 PMC150107

[20c] A. Henne , R. Daniel , R. A. Schmitz , G. Gottschalk , Appl. Environ. Microbiol. 1999, 65, 3901–3907. 10473393 10.1128/aem.65.9.3901-3907.1999 PMC99718

[21] P. Lorenz , J. Eck , Nat. Rev. Microbiol. 2005, 3, 510–516. 15931168 10.1038/nrmicro1161

[22a] N. Itoh , K. Isotani , Y. Makino , M. Kato , K. Kitayama , T. Ishimota , Enzyme Microb. Technol. 2014, 55, 140–150; 24411457 10.1016/j.enzmictec.2013.10.012

[22b] N. Itoh , M. Kazama , N. Takeuchi , K. Isotani , J. Kurokawa , FEBS Open Bio 2016, 6, 566–575; 10.1002/2211-5463.12067 PMC4887972 27419059

[22c] J. W. E. Jeffries , N. Dawson , C. Orengo , T. S. Moody , D. J. Quinn , H. C. Hailes , J. M. Ward , ChemistrySelect 2016, 1, 2217–2220.

[23] G. Qu , B. Liu , Y. Jiang , Y. Nie , H. Yu , Z. Sun , Bioresources Bioprocessing 2019, 6, 18.

[24a] A. Huebner , L. F. Olguin , D. Bratton , G. Whyte , W. T. Huck , A. J. de Mello , J. B. Edel , C. Abell , F. Hollfelder , Anal. Chem. 2008, 80, 3890–3896; 18399662 10.1021/ac800338z

[24b] R. Ostafe , R. Prodanovic , W. Lloyd Ung , D. A. Weitz , R. Fischer , Biomicrofluidics 2014, 8, 041102. 25379082 10.1063/1.4886771 PMC4189127

[25] G. Woronoff , A. El Harrak , E. Mayot , O. Schicke , O. J. Miller , P. Soumillion , A. D. Griffiths , M. Ryckelynck , Anal. Chem. 2011, 83, 2852–2857. 21413778 10.1021/ac200373n

[26] T. Beneyton , S. Thomas , A. D. Griffiths , J. M. Nicaud , A. Drevelle , T. Rossignol , Microb. Cell Fact. 2017, 16, 18. 28143479 10.1186/s12934-017-0629-5 PMC5282883

[27] S. S. Terekhov , I. V. Smirnov , A. V. Stepanova , T. V. Bobik , Y. A. Mokrushina , N. A. Ponomarenko , A. A. Belogurov, Jr. , M. P. Rubtsova , O. V. Kartseva , M. O. Gomzikova , A. A. Moskovtsev , A. S. Bukatin , M. V. Dubina , E. S. Kostryukova , V. V. Babenko , M. T. Vakhitova , A. I. Manolov , M. V. Malakhova , M. A. Kornienko , A. V. Tyakht , A. A. Vanyushkina , E. N. Ilina , P. Masson , A. G. Gabibov , S. Altman , Proc. Natl. Acad. Sci. USA 2017, 114, 2550–2555. 28202731 10.1073/pnas.1621226114 PMC5347554

[28] M. Klaus, G. A. Aleku, S. Neun, K. Neufeld, F. Hollfelder, unpublished results.

[29] E. A. Legenzov , N. D. Dirda , B. M. Hagen , J. P. Kao , PLoS One 2015, 10, e0133518. 26186650 10.1371/journal.pone.0133518 PMC4505926

[30] O. S. Wolfbeis , E. Fürlinger , H. Kroneis , H. Marsoner , Fresenius Z. Anal. Chem. 1983, 14, 119–124.

[31] P. J. Zurek , R. Hours , U. Schell , A. Pushpanath , F. Hollfelder , Lab Chip 2021, 21, 163–173. 33242058 10.1039/d0lc00830c

[32] M. Naumann, J. S. Wooten, **2013**, Patent WO 2013/139673 Al.

