# Supplementary material for: Ultrahigh‐Throughput Detection of Enzymatic Alcohol Dehydrogenase Activity in Microfluidic Droplets with a Direct Fluorogenic Assay
Source: Chembiochem. 2021 Oct 13;22(23):3292–9. doi: 10.1002/cbic.202100322 (PMC9291573; doi:10.1002/cbic.202100322)
Supplement: Supplementary file 1 — Supporting Information [file CBIC-22-3292-s001.pdf]

ChemBioChem

Supporting Information

Ultrahigh-Throughput Detection of Enzymatic Alcohol Dehydrogenase Activity in Microfluidic Droplets with a Direct Fluorogenic Assay

Miriam Klaus[+], Paul Jannis Zurek[+], Tomasz S. Kaminski, Ahir Pushpanath, Katharina Neufeld,* and Florian Hollfelder*

Table of Contents

	Page
**1. Supplementary Figures**	S3
Figure S1. Long-term retention of reaction product 7 and substrate	S3
Figure S2. Microfluidic device used for model selections and the determination of enrichment values	S4
**2. Consideration of Detection Limits**	S5
Table S1: Comparison of the detection limits of alternative detection modes for redox reactions of ketone groups	
**3. Materials and General Instrumentation**	S6
**4. Synthetic Procedures**	S6
**5. Optical Characterization of Pyranine and Fluorogenic ADH Substrates**	S11
**6. Fluorophore retention experiments in droplets**	S11
**7. ADH Assays with Substrates 4, 7 and 10 in Droplets**	S12
7.1 Chip design and preparation of microfluidic devices	
7.2 Fluorescence-activated Droplet Sorting	
7.3 DNA Recovery	
**8. Practical Note on Maximising the Pyranine Fluorescence Readout Simultaneously with Efficient Cell Lysis**	S14
**9. ADH Assays with Substrates 4, 7 and 10 in Microtiter Plates**	S15
**10. Primary data for the calculation of enrichment values**	S16
**11. NMR spectra**	S17
**12. Supplementary References**	S21

1. Supplementary Figures

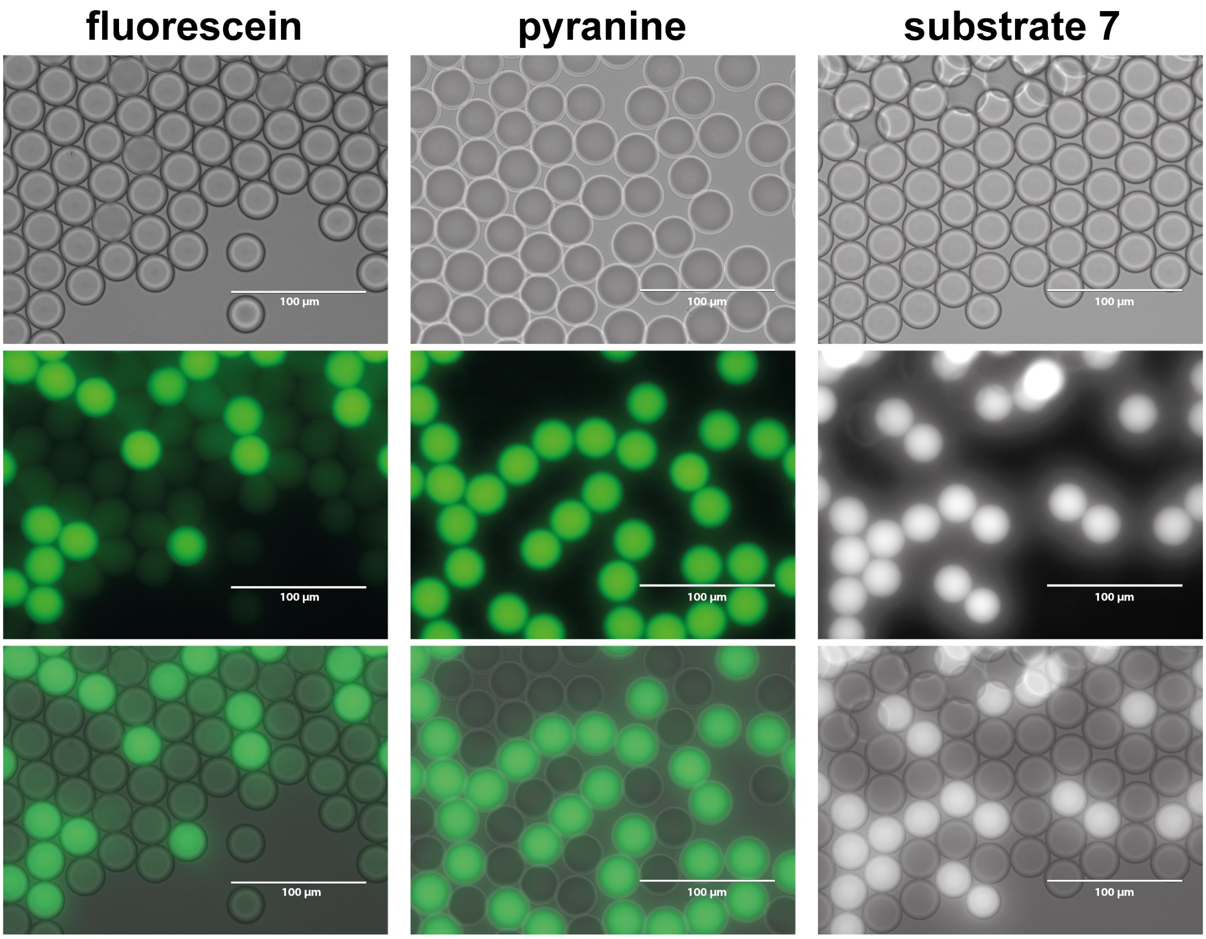

Figure S1. Long-term retention of the reaction product, pyranine **1** (*centre*, Fig. 2), substrate **7** (*right*, Fig. 2) and, as a control, fluorescein. A mixed population of 20 pL droplets containing the analytes (fluorescein: 10 µM; **1**: 10 µM; **7**: 300 µM) or buffer only, were generated, mixed and incubated for six weeks. The droplets were visualised in brightfield (top row), by fluorescence detection (middle row) and shown as an overlay of brightfield and fluorescence images. Fluorescein and pyranine **1** were detected in the GFP channel (ex. 482/25 em. 524/24), while the fluorescence of substrate **7** was detected in the DAPI channel (ex. 357/44 em. 447/60). Under these conditions fluorescein is shown to leak (as evidenced by an increase in fluorescence in the originally non-fluorescent droplets, ~28% leakage), while for **1** and **7**, the original distinction between droplets with and without analyte is maintained (2% and 6% leakage, respectively). Thus leakage of pyranine is greatly reduced compared to fluorescein, even for this extended time interval, suggesting that slow reactions can be followed over timescales of weeks. The ability to detect small amounts of product will allow identification of enzymes with relatively weak turnover. *Scale bar:* 100 µm. *Conditions*: 50 mM sodium phosphate buffer pH 8, room temperature (25 °C).

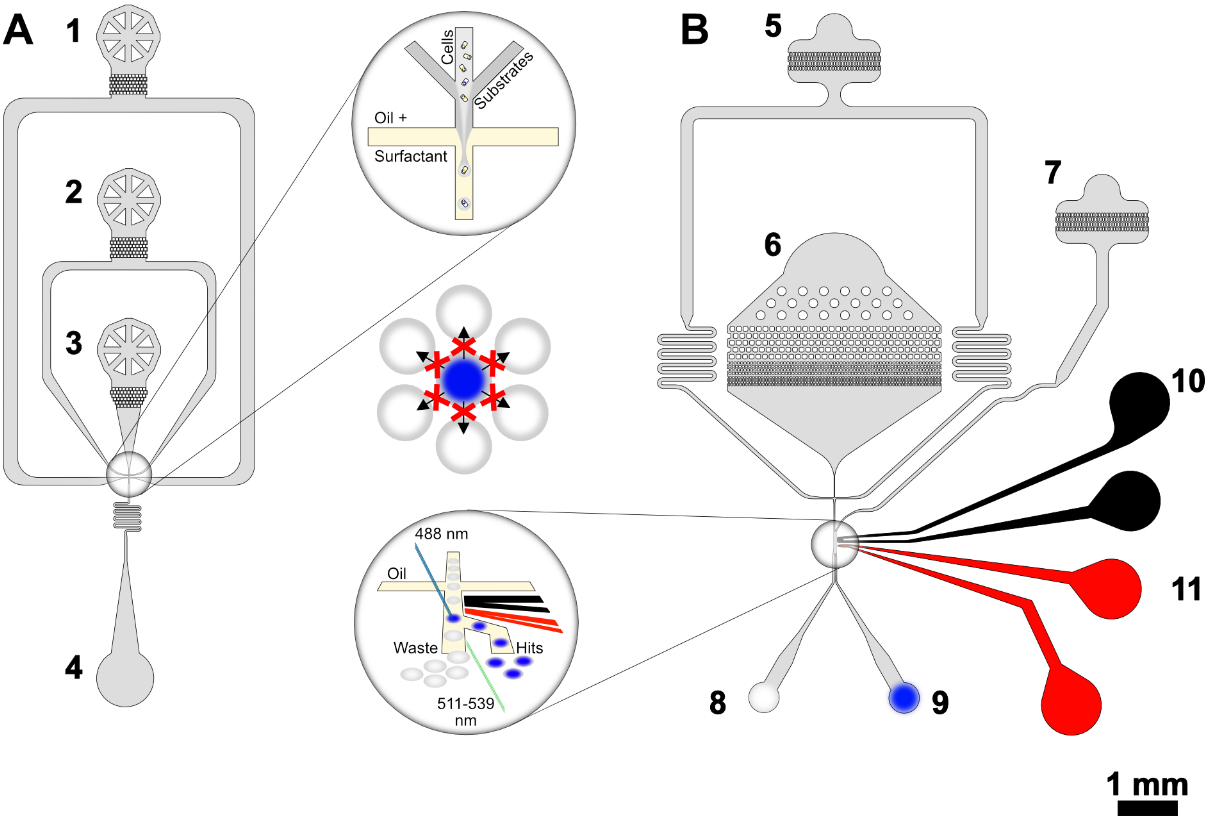

Figure S2. Microfluidic device used for model selections and the determination of enrichment values. Gray colour denotes fluidic channels, red colour indicates a signal electrode and black colour the ground electrode. **(A)** *Droplet co-compartmentalisation of substrate and cells.* A device featuring a flow focusing junction (with channel dimensions 16 × 12 µm, width × depth) was used for the generation of 3 pL monodisperse water-in-oil droplets. Inlet 1 was used for providing the fluorinated oil with admixed surfactant. The two aqueous solutions, containing the substrate and the bacteria were injected from inlets 2 and 3, respectively. Droplets leave the microfluidic device through outlet 4 and were collected in a LoBind Eppendorf tube, where they were stored to allow for the enzymatic reaction. **(B)** *Fluorescence-activated droplet sorting (FADS)* was performed in a device with a 40 × 22 µm (width × depth) sorting junction. Inlet 5 supplies spacing oil and inlet 6 the emulsion droplets. Additional bias oil was delivered using inlet 7. The outlets for collection of the negative and positive droplets are marked by 8 and 9, respectively. The dielectrophoretic force was generated with "salt-water electrodes" [1], filled with 5 M NaCl solution deposited in the chip prior to the experiment using inlets 10 and 11 for ground and signal electrodes, respectively. *Scale bar*: 1 mm

2. Consideration of Detection Limits

Table S1: Comparison of the detection limits of alternative detection modes for redox reactions of ketone groups, monitored in microfluidic droplets.

**Detection mode**	**Analyte**	**Sensitivity**	**Droplet volume**	**Detection limit** [molecules per droplet][a]	**Turnovers per enzyme molecule** (single cell)[b]	**Reference**
Fluorescence intensity	Pyranine **1**	<0.1 µM	3 pL	1.8×10^{5}	0.22	This study
Absorbance	NAD(P)H / WST-1	10 µM	180 pL	1.1×10^{9}	1350	[2]
Electro-chemistry	NAD(P)H	1 µM	30 nL	1.8×10^{10}	22600	[3]
Mass spectrometry	1-(imidazo [2,1-b] thiazol-6-yl) propan-2-amine	30 µM	25 nL	4.5×10^{11}	565000	[4]

[a] Avogadro constant 6.022×10^{23}
[b] Assuming expression of 8×10^{5} enzyme molecules per *E. coli* cell [2]

3. Materials and general instrumentation

All chemicals and solvents were purchased from Sigma-Aldrich, Acros Organics, or Alfa Aesar and used as received without further purification. Thin layer chromatography was performed using Polygram SIL G UV254 TLC sheets from Macherey-Nagel and UV detection. Column chromatography was performed using Silica Gel 60 M from Macherey-Nagel with a particle size of 0.04-0.063 mm. NMR spectra were recorded on a Bruker DRX-400 spectrometer at 400 MHz for ^{1}H-NMR and at 100 MHz for ^{13}C-NMR. Chemical shifts (δ values) in ^{1}H- and ^{13}C-NMRs were calibrated to the residual solvent resonance at 7.26 and 77.16 ppm for $CDCl_3$ and 2.5 and 39.52 ppm for DMSO-d6, respectively. Mass spectra were obtained using a Micromass LCT spectrometer (Waters, Milford, MA, USA) in ESI negative mode. UV-visible spectra and optical assays were measured on an M1000 Tecan UV/VIS-spectrophotometer.

4. Synthetic Procedures

4-tosyloxy-2-butanol (6)

A solution of 1,3-butandiol (**5**) (2.000 g, 22.2 mmol, 1.0 eq.) in dry pyridine (10 mL) was cooled in an ice bath and *p*-toluenesulfonyl chloride (4.656 g, 24.4 mmol, 1.1 eq.) was added. The reaction was allowed to warm up to RT and stirred for an additional 30 min. The sample was diluted with H_2O (50 mL) and extracted with Et_2O (3x70 mL). The combined organic phases were washed with 1 M aq. CuSO4 (2x50 mL), dried with $MgSO_4$, and concentrated *in vacuo*. Chromatography of the crude product on silica gel with EtOAc/hexane (30:70→70:30) afforded the desired product **6** (2.862 g, 11.7 mmol, 53%) as a colourless oil.

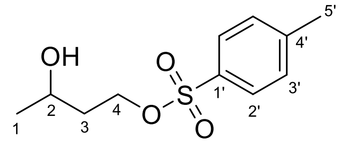

^{1}H-NMR ($CDCl_3$): 1.14 (d, $^3J_{1,2}$ = 6.1 Hz, 3H, 1-H), 1.61−1.73 (m, 1H, 3-H_a), 1.73−1.84 (m, 1H, 3-H_b), 2.12 (s, 1H, OH), 2.42 (s, 3H, 5′-H), 3.89 (m_c, 1H, 2-H), 4.04–4.12 (m, 1H, 4-H_a), 4.16–4.24 (m, 1H, 4-H_b), 7.32 (d, $^3J_{3',2'}$ = 8.1 Hz, 2H, 3′-H), 7.76 (d, $^3J_{2',3'}$ = 8.1 Hz, 2H, 2′-H). ^{13}C-NMR ($CDCl_3$): 21.7 (C-5′), 23.6 (C-1), 37.9 (C-3), 64.1 (C-2), 68.0 (C-4), 127.9 (C-2′), 130.0 (C-3′), 133.0 (C-1′), 144.9 (C-4′). LC-MS (ESI, negative ion): m/z (%) = 264 (100) [$(M-2H)^{2-}$], 529 (12) [$(M-H)^-$]. Rf: AcOEt/hexane (70:30) = 0.68.

3-iodo-1-phenylpropan-1-ol (9)

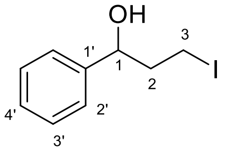

Sodium iodide (2.788 g, 18.6 mmol, 5.0 eq.) was added to a solution of 3-chloro-1-phenyl-1-propanol **8** (634 mg, 3.72 mmol, 1.0 eq.) in acetone (20 ml). The mixture was heated to reflux at 60°C overnight. After the solution was cooled to RT, the solvent was evaporated *in vacuo*. The residue was dissolved in water (20 mL) and extracted with diethyl ether (3x 50 mL), the organic phases were combined, washed with brine, dried over $MgSO_4$ and evaporated *in vacuo* to provide the desired product **9** (913 mg, 3.48 mmol, 94%) as a white solid.

^{1}H-NMR ($CDCl_3$): 7.35–7.38 (m, 4H, 2′-H, 3′-H), 7.31 (m_c, 1H, 4′-H), 4.82 (m_c, 1H, 1-H), 3.27–3.36 (m, 1H, 3-H_a), 3.14–3.23 (m, 1H, 3-H_b), 2.11–2.32 (m, 2H, 2-H), 2.00 (s, 1H, OH). ^{13}C-NMR ($CDCl_3$): 2.71 (C-3), 42.5 (C-2), 74.3 (C-1), 126.0 (C-2′), 128.1 (C-4′), 128.8 (C-3′), 143.6 (C-1′). LC-MS (ESI, negative ion): m/z (%) = 127 (100) [$(I)^-$], 529 (12) [$(M-H)^-$].

8-{3-[(tetrahydro-2H-pyran-2-yl)oxy]propyl}-1,3,6-pyranine (3)

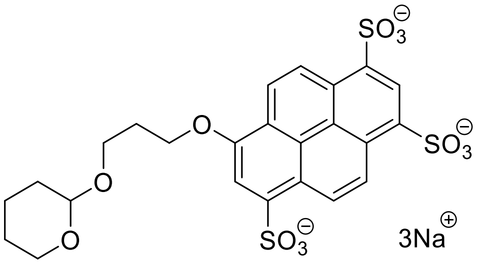

Based on the procedure of Naumann and Wooten[5] pyranine (500 mg, 0.95 mmol, 1.0 eq.) was dissolved in DMSO (15 mL) by vigorous mixing for 2 h, after which 50 wt-% aq. NaOH solution (88 µL, 1.68 mmol, 1.8 eq.) was added and stirring was continued for another 30 min. Then, in one portion, 1-bromo-3-(tetrahydro-pyranyloxy)propane **2** (379 mg, 1.70 mmol, 1.8 eq.) was added and the reaction mixture was stirred for 2 d at RT until green fluorescence disappeared from the reaction mixture. Acetone (80 mL) was added to the reaction mixture in order to precipitate the desired reaction product. After centrifugation (4000 rpm, RT, 5 min) the supernatant was removed and the precipitate was re-dissolved in a minimal amount of water possible. The obtained product was precipitated again in acetone (40 mL) and obtained after another centrifugation step and removal of the supernatant. The obtained pellet was washed twice with acetone (2x20 mL) via vortexing and separation of liquid by centrifugation. Subsequently, the obtained pellet was dissolved in water (20 mL) and freeze-dried to give the desired product **7** (290 mg, 0.44 mmol, 46%) as a bright orange powder.

^{1}H-NMR (DMSO-d6): δ = 1.46 (4H, m), 1.63 (1H, m), 1.73 (1H, m), 2.22 (2H, s, $ArOCH_2C\underline{H}_2$), 3.72 (2H, m), 3.98 (1H, s), 4.44 (2H, s, $ArOC\underline{H}_2CH_2$), 4.64 (1H, s, C<u>H</u>O), 8.21 (1H, s, ArH), 8.38 (1H, d, ArH), 8.95 (1H, d, ArH), 9.04 (2H, m, ArH), 9.12 (1H, d, ArH); ^{13}C-NMR (DMSO-d6): δ = 19.2, 25.0, 29.4 ($ArOCH_2\underline{C}H_2$), 30.3, 61.3 ($\underline{C}H_2OCH$), 63.4

f($\underline{C}H_2OCH$), 65.8 ($ArO\underline{C}H_2CH_2$), 98.1 (CHO), 108.9 (ArCH), 119.7 (ArCH), 120.2 (Ar), 120.5 (Ar), 123.6 (ArCH), 124.5 (ArCH), 125.1 (Ar), 125.2 (Ar), 125.9 (ArCH), 126.4 (ArCH), 127.7 (Ar), 128.1 (Ar), 139.5 ($ArCSO_3$), 139.7 ($ArCSO_3$), 143.2 ($ArCSO_3$), 151.5 (ArCO);
MS (ESI-): *m/z* calc. 600.0430, found *m/z* (%) = 299 (100) [$(M{-}2H)^{2-}$], 599 (7) [$(M{-}H)^{-}$].

8-(3-hydroxypropoxy)-1,3,6-pyranine (4)

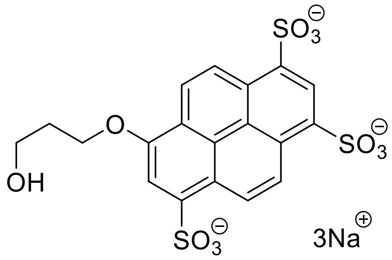

Procedure 1: 8-{3-[(tetrahydro-2H-pyran-2-yl)oxy]propyl}-1,3,6-pyranine (**3**) (188 mg, 0.28 mmol, 1.0 eq.) was dissolved in methanol/water (95:5, 18 mL), *para*-toluenesulfonic acid (21 mg, 0.11 mmol, 0.4 eq.) was added and stirring was continued overnight. Subsequently, methanol was removed *in vacuo* and acetone (40 mL) was added to the remaining reaction mixture in order to precipitate the desired reaction product. After centrifugation (4000 rpm, RT, 5 min) the supernatant was removed and the precipitate was re-dissolved in a minimal amount of water possible. The obtained product was precipitated again in acetone (40 mL) and obtained after another centrifugation step and removal of the supernatant. The obtained pellet was washed twice with acetone (2x20 mL) via vortexing and separation of liquid by centrifugation. Subsequently, the obtained pellet was dissolved in water (20 mL) and freeze-dried to give the desired product **4** (125 mg, 0.21 mmol, 75%) as a bright yellow powder with NMR purity.

Procedure 2: Based on a patented procedure[5], pyranine (5.0 g, 9.54 mmol, 1.0 eq.) was dissolved in DMSO (150 mL) by vigorous mixing for 2 h, after which 50 wt-% aq. NaOH solution (880 µL, 16.8 mmol, 1.8 eq.) was added and stirring was continued for another 30 min. Then, in one portion, 3-bromo-1-propanol **11** (2.58 mL,) was added and the reaction mixture was stirred for 4 d at RT until green fluorescence disappeared from the reaction mixture. Acetone (800 mL) was added to the reaction mixture in order to precipitate the desired reaction product. After centrifugation (4000 rpm, RT, 5 min) the supernatant was removed and the precipitate was re-dissolved in a minimal amount of water possible. The obtained product was precipitated again in acetone (400 mL) and obtained after another centrifugation step and removal of the supernatant. The obtained pellet was washed twice with acetone (2x200 mL) via vortexing and separation of liquid by centrifugation. Subsequently, the obtained pellet was dissolved in water (100 mL) and freeze-dried to give the desired product **4** (3.806 g, 6.53 mmol, 68%) as a bright yellow powder.

^{1}H-NMR (DMSO-d6): δ = 2.16 (2H, dd, $OCH_2C\underline{H}_2$), 3.79 (2H, s, $C\underline{H}_2OH$), 4.48 (2H, s, $OC\underline{H}_2CH_2$), 4.74 (1H, s, OH), 8.26 (1H, s, ArH), 8.42 (1H, d, ArH), 9.00 (1H, d, ArH), 9.08 (2H, m, ArH), 9.16 (1H, d, ArH); ^{13}C-NMR (DMSO-d6): δ = 33.3 ($OCH_2\underline{C}H_2$), 58.4 (CH_2OH), 66.8 ($O\underline{C}H_2CH_2$), 109.9 (ArCH), 120.6 (Ar), 121.2 (Ar), 121.3 (ArCH), 124.5 (ArCH), 125.4 (ArCH), 126.1 (Ar), 126.4 (Ar), 126.9 (ArCH), 127.3 (ArCH), 128.6 (Ar), 129.0 (Ar), 140.4 ($ArCSO_3$), 140.6 ($ArCSO_3$), 143.5 ($ArCSO_3$), 152.5 (ArCO); MS (ESI-): *m/z* calc. 515.9588, found *m/z* (%) = 257 (100) [$(M{-}2H)^{2-}$], 515 (17) [$(M{-}H)^{-}$].

8-(3-hydroxybutyl)-1,3,6-pyranine (7)

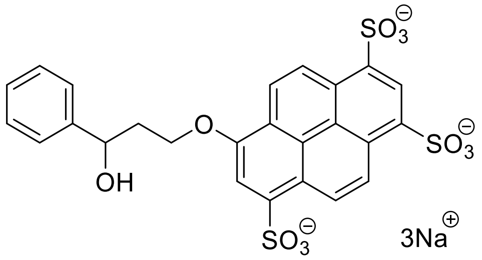

Based on a patented procedure[5], pyranine (500 mg, 0.95 mmol, 1.0 eq.) was dissolved in DMSO (15 mL) by vigorous mixing for 2 h, after which 50 wt-% aq. NaOH solution (88 µL, 1.68 mmol, 1.8 eq.) was added and stirring was continued for another 30 min. Then, in one portion, tosylate **6** (464 mg, 1.90 mmol, 2.0 eq.) was added and the reaction mixture was stirred for 2 d at RT until green fluorescence disappeared from the reaction mixture. Acetone (80 mL) was added to the reaction mixture in order to precipitate the desired reaction product. After centrifugation (4000 rpm, RT, 5 min) the supernatant was removed and the precipitate was re-dissolved in a minimal amount of water possible. The obtained product was precipitated again in acetone (40 mL) and obtained after another centrifugation step and removal of the supernatant. The obtained pellet was washed twice with acetone (2x20 mL) via vortexing and separation of liquid by centrifugation. Subsequently, the obtained pellet was dissolved in water (20 mL) and freeze-dried to give the desired product **7** (396 mg, 0.66 mmol, 69%) as a bright orange powder. **7** was pure by NMR analysis, but the small pH-dependent increase in fluorescence at 515 nm (Figure 2a, lower panel) suggests the presence of small remaining amounts (<5%) of pyranine.

^{1}H-NMR (DMSO-d6): δ = 2.12 (2H, s, $OCH_2C\underline{H_2}$), 4.39 (1H, s, $OC\underline{H_2}CH_2$), 4.53 (1H, s, $OC\underline{H_2}CH_2$), 5.04 (1H, s, C$\underline{H}$OH), 5.55 (1H, s, OH), 7.29 (1H, s, PhH), 7.39 (2H, s, PhH), 7.48 (2H, s, PhH), 8.23 (1H, s, ArH), 8.43 (1H, d, ArH), 8.99 (1H, m, ArH), 9.08 (2H, m, ArH), 9.17 (1H, d, ArH); ^{13}C-NMR (DMSO-d6): δ = 40.0 ($OCH_2\underline{C}H_2$), 66.8 ($O\underline{C}H_2CH_2$), 70.3 (CHOH), 109.8 (ArCH), 120.6 (Ar), 121.3 (Ar), 121.4 (ArCH), 124.5 (ArCH), 125.4 (ArCH), 126.1 (Ar), 126.4 (Ar), 126.8 (PhCH), 126.9 (ArCH), 127.3 (ArCH), 127.8 (PhCH), 128.6 (Ar), 129.0 (Ar), 129.1 (PhCH), 140.4 (PhCH), 140.6 ($ArCSO_3$), 144.1 ($ArCSO_3$), 146.8 ($ArCSO_3$), 152.5 (ArCO); MS (ESI-): *m/z* calc. 592.0168, found *m/z* (%) = 295 (100) [$(M-2H)^{2-}$], 591 (9) [$(M-H)^{-}$].

8-(3-hydroxy-3-phenylpropoxy)-1,3,6-pyranine (10)

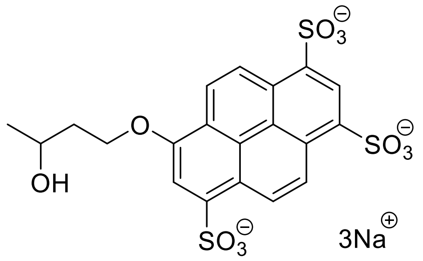

Based on a the procedure of Naumann and Wooten[5] pyranine (519 mg, 0.99 mmol, 1.0 eq.) was dissolved in DMSO (15 mL) by vigorous mixing for 2 h, after which 50 wt-% aq. NaOH solution (91 µL, 1.73 mmol, 1.7 eq.) was added and stirring was continued for another 30 min. Then, in one portion, 3-iodo-1-phenylpropan-1-ol **9** (520 mg, 1.98 mmol, 2.0 eq.) was added and the reaction mixture was stirred for 2 d at RT until green fluorescence disappeared from the reaction mixture. Acetone (80 mL) was added to the reaction mixture in order to precipitate the desired reaction product. After centrifugation (4000 rpm, RT, 5 min) the supernatant was removed and the precipitate was re-dissolved in a minimal amount of water possible. The obtained product was precipitated again in acetone (40 mL) and obtained after another centrifugation step and removal of the supernatant. The obtained pellet was washed twice with acetone (2x20 mL) via vortexing and separation of liquid by centrifugation. Subsequently, the obtained pellet was dissolved in water (20 mL) and freeze-dried to give the desired product **10** (432 mg, 0.65 mmol, 66%) as a bright orange powder with NMR purity.

^{1}H-NMR (DMSO-d6): δ = 1.22 (3H, s, CH_3), 2.03 (2H, d, $OCH_2C\underline{H}_2$), 4.03 (1H, s, C<u>H</u>OH), 4.44 (2H, s, $OC\underline{H}_2CH_2$), 4.74 (1H, s, OH), 8.22 (1H, s, ArH), 8.38 (1H, d, ArH), 8.95 (1H, d, ArH), 9.04 (2H, m, ArH), 9.12 (1H, d, ArH); ^{13}C-NMR (DMSO-d6): δ = 25.0 (CH_3), 39.4 ($OCH_2\underline{C}H_2$), 64.1 (CHOH), 67.0 ($O\underline{C}H_2CH_2$), 109.9 (ArCH), 120.6 (Ar), 121.2 (Ar), 121.3 (ArCH), 124.5 (ArCH), 125.4 (ArCH), 126.1 (Ar), 126.4 (Ar), 126.8 (ArCH), 127.3 (ArCH), 128.6 (Ar), 129.0 (Ar), 140.4 ($ArCSO_3$), 140.6 ($ArCSO_3$), 144.1 ($ArCSO_3$), 152.3 (ArCO); MS (ESI-): *m/z* calc. 530.0011, found *m/z* (%) = 264 (100) [$(M{-}2H)^{2-}$], 529 (12) [$(M{-}H)^{-}$].

5. Optical characterization of pyranine and fluorogenic ADH substrates

In order to achieve optical characterization of compounds of interest, pyranine **1** or pyranine substrates **4**, **7**, and **10** were combined with buffers of different pH values. Fluorescence excitation and emission scans were performed to characterize the generated substrates and compare obtained values to those of 'free' pyranine.

Table S2: Experimental set-up for optical properties of substrates **4**, **7** and **10**.

compound	stock solution		∑ 100 µL	final concentration
pyranine **1** or substrate **4**, **7** or **10** in water	100 µM	10×	10 µL	10 µM
different buffers[1]	400 mM	4×	25 µL	100 mM
milliQ H_2O	-	-	65 µL	-

measurements:

sample set-up in a MTP; fluorescence and emission scans were performed

[1] sodium acetate buffers with pH values between 4.0-5.5; potassium phosphate buffers with pH values between 5.5-8.0 and 11.0-11.9; Tris/HCl buffers with pH values between 8.0-9.0; and sodium carbonate buffers with pH values between 9.2-10.8

6. Fluorophore retention experiments in droplets

The retention of fluorophores in droplets was determined by fluorescence microscopy (EVOS FL, Thermo Fisher). Droplets containing 10 µM fluorophore (pyranine **1**, fluorescein or resorufin) or 300 µM fluorophore (pyranine substrate **7**) were generated in 50 mM buffer at different pH values (sodium citrate pH 4, sodium phosphate pH 6 and pH 8, sodium carbonate pH 10) and mixed 1:1 with droplets containing no fluorophore. Droplet generation was performed in a conventional flow focussing device (12 µm height, 16 µm width, Fig. S1) with HFE-7500 (3M Novec) containing 1% 008-Fluorosurfactant (RAN Biotechnologies) as oil phase, to a volume of 20 pL. Droplets were collected and incubated in the dark at room temperature. Fluorescence microscopy images in the GFP channel (ex. 482/25 em. 524/24, used for pyranine **1** and fluorescein), the DAPI channel (ex. 357/44 em. 447/60, used for pyraninie substrate **7**) or the RFP channel (ex. 542/20 em. 593/40, used for resorufin) were obtained (Fig. S2) and used to calculate fluorophore leakage between droplets (Fig. 2d). Leakage was calculated as follows: The image was converted to grey scale and intensities were normalized from 0 to 255. Average grey values of 10 empty and 10 filled droplets were obtained from the mixture, as well as a large portion of the background intensity. Background grey values were subtracted, and leakage calculated as the proportion of average intensity of empty droplets to average intensity of full droplets, yielding 100% leakage if all droplets show the same fluorescence intensity and 0% leakage if empty droplets are not discernible from background intensity.

7. ADH assays with substrates 4, 7 and 10 in droplets

7.1 Chip design and preparation of microfluidic devices

The microfluidic devices with rectangular channels for droplet generation and droplet pico-injection were fabricated following standard photolithography and soft lithography protocols.[6]

7.1.1. Photolithography protocol

First, microfluidic moulds were patterned on 3” size silicon wafer (Prime CZ-Si, single side polished, purchased from Microchemicals, Germany) using high-resolution film masks (Microlithography Services Ltd, UK). Photomasks of devices were designed in AutoCAD 2018 or DraftSight (Dassault Systems) and are available at http://openwetware.org/wiki/DropBase. The designs are also attached to this manuscript as a supplementary DXF file entitled ‘SI_2_CAD designs of devices’.

MJB4 mask aligner (SUSS MicroTec, Germany) was used to UV-expose of all the wafers spin-coated with a layer of SU-8 2010 or SU-8 2025 photoresists (Kayaku Advanced Materials, Japan). The thickness of the channels was measured using a DektakXT Stylus profilometer (Bruker, USA).

Table S3. Features of device designs used in this study.

	Device type	
	Flow focusing droplet generation device	Bias oil FADS device
Nominal thickness	12 µm	20 µm
Photoresist used	Su8-2010	Su8-2025
Spin coating speed	1st step: 10 sec, 500 rpm<br>2nd step: 30 sec, 2000 rpm	1st step: 10 sec, 500 rpm<br>2nd step: 30 sec, 4000 rpm
Pre-baking	3 min at 95 ºC	1 min at 65 ºC<br>5 min at 95 ºC
Exposure (at ~10 mW cm^{-2})	2 x 6 sec, 2 sec wait time	2 x 7 sec, 2 sec wait time
Post-baking	3 min at 95 ºC	1 min at 65 ºC<br>5 min at 95 ºC
Development in a beaker filled with 30-50 mL of PGMEA (propylene glycol methyl ether acetate, Sigma Aldrich)	Approx. 5 min until all uncured Su-8 is removed from the wafer, development time depends on the intensity of manual agitation	
Hard baking	10 min at 200 ºC	
Measured range of thicknesses	11.8-11.9 µm	21.7-21.8 µm

7.1.2. Soft lithography protocol

To make a single PDMS [poly(dimethyl siloxane)] microfluidic device, 20-30 grams of silicone elastomer base and the curing agent (Sylgard^{TM} 184, Dow Corning, USA) were mixed in the a 10:1 (w/w) ratio in a plastic cup and next degassed in a vacuum chamber. PDMS was then poured on the SU-8 master wafer and cured in the oven at 65 ºC for at least 4 h. Next, the inlet holes were punched using a 1 mm diameter biopsy punch with plungers (Kai Industries Co. Ltd., Japan). The patterned flow-focusing chip was then plasma-bonded to a 50 mm x 75 mm x 1 mm (length x width x thickness) glass slide (VWR), while the droplet sorting chip was bonded to a 24 mm x 50 mm x 0.13 mm coverslip (Agar Scientific). All bonding procedures were performed in a low-pressure oxygen plasma generator (Femto, Diener Electronics, Germany). Next, the hydrophobic modification of microfluidic channels was performed by flushing the device with 1% (v/v) trichloro(1H,1H,2H,2Hperfluorooctyl)-silane (Sigma Aldrich) in HFE-7500 (3M) and baked on a hot plate at 75 °C for at least 30 minutes in order to evaporate the fluorocarbon oil and silane mix.

7.2 Fluorescence-activated droplet sorting

Droplets were generated by co-encapsulating cell suspension and reaction mix to 3 pL droplets. To achieve encapsulation of single cells, an induced culture expressing ADH or a negative control was diluted in buffer to a density allowing the encapsulation of ~0.2 cells per droplet. Co-encapsulation with reaction mix leads to the lysis of the cell, releasing the enzyme, leading to reaction progress and release of free pyranine.

Water-in-oil droplets were re-injected into the sorting device at around 20 μL/h and spaced out with oil (1% w/v 008-FluoroSurfactant in HFE-7500) at 200 μL/h. Additionally, 100 μL/h bias oil was flown in at the sorting junction to prevent accidental spill-over of droplets into the collection channel (Fig. S1B). Droplets were sorted based on previous protocols[7] at frequencies of up to 600 Hz. A 488-nm laser was focused upstream of the sorting junction through a 40x microscope objective (LUCPlanFLN, Olympus) for fluorophore excitation and the emitted fluorescent light was collected and amplified using a photomultiplier tube (PMM02, Thorlabs). If fluorescence exceeded a user-defined threshold, the pulse and function generators were triggered to generate a square pulse of 50-500 μs at 4-8 Vpp. This pulse was next amplified 1000-fold by a high-voltage amplifier (610E, Trek) and applied on the sorting device via salt-water electrodes (5 M NaCl), dragged the selected droplet away from the path of least resistance (a wide waste channel) into the collection channel. The sorting events were recorded with a fast camera (Phantom Miro eX4) to allow analysis of whether the desired droplets with increased fluorescence were faithfully selected after the sort was carried out. Optical inspection of the movies thus recorded provided confirmation that only single droplets were selected for each pulse.

7.3 DNA recovery

To recover plasmid DNA, sorted droplets were collected into a 1.5 mL low DNA retention reaction tube (DNA LoBind, Eppendorf). The sample was de-emulsified by adding 50-200 μL 1H,1H,2H,2H-perfluorooctanol (97%, Alfa Aesar) and 100 μL of a 0.2-2 ng/μL salmon sperm DNA solution (in nuclease-free water; Invitrogen). The content of the tube was vigorously mixed by vortexing for one minute and phase separation was achieved by centrifugation for 1 min at 2,000 rpm at room temperature. The aqueous layer was transferred to a fresh reaction

tube and the oil extraction was repeated twice. After phase separation, DNA was extracted from the aqueous phase using a DNA purification kit (Clean & Concentrator-5, Zymo Research). Recovered plasmid DNA was then transformed into *E. coli* cells (E. cloni 10G Elite, Lucigen) and ADH activity was verified in microtiter plates as described below.

Table S4: Typical quantities for ADH assays in droplets.

	compound	stock solution		Σ 1 mL	final concentration
reaction mix aqueous phase 1	rLysozyme	-	-	0.4 - 4 µL	0.2 - 2 µL/mL
	Polymyxin B in buffer	1 mM	10x	200 µL	100 µM
	$NADP^+$ in buffer	6 mM	10x	200 µL	600 µM
	Substrate **4** in buffer	2 mM	20x	300 µL	300 µM
	100 mM KPi-buffer, pH 8.0	-	-	To 100 µL	-
cell suspension aqueous phase 2	ADH expressing *E. coli*[1]	OD_{600} = 0.8		500 µL	
	100 mM KPi-buffer, pH 8.0	-		250 µL	OD_{600} = 0.4 (stock)
	Percoll	4×		250 µL	OD_{600} = 0.2 (droplet) λ = ~ 0.3 (based on 3 pL droplets)
oil phase	HFE-7500 3M[2]	-		-	pure
	HFE + 1.5% (w/v) RAN	HFE + 10% (w/v) RAN		8.5 mL HFE + 1.5 mL stock	1.5% (w/v)

[1] 1 mL of cells was harvested (1 min, 5000 rpm); pellet was resuspended in and collected from 1 mL buffer; pellet was resuspended in 1 mL buffer, further diluted to OD_{600} = 0.8 and used for the experiment
[2] Reaction mix and oil was sterile filtered before application.

8. Practical note on maximising the pyranine fluorescence readout simultaneously with efficient cell lysis

When CelLytic B was used as a lysis reagent in initial experiments, no pyranine fluorescence could be observed in droplets, because the signal maxima had shifted (for excitation and emission to 380 nm and 525 nm, respectively). An exploration of other possible lysis reagents suggested a combination of rLysozyme (heterologously produced lysozyme, Merck) and polymyxin B as a useful compromise between the somewhat conflicting goals of ensuring high fluorescence intensity and efficient lysis at the same time. The fluorescence intensities of droplets were monitored by excitation at 488 nm. A positive droplet population showing ADH activity clearly demarcated from the negative/empty droplet population with low variability in their signal distributions (Fig. 4).

9. ADH assays in microtiter plates

Verification of 'hits' for enrichment experiments was performed in 96-well microtiter plates in a secondary assay. The substrates **4** or **7** were combined with buffer, ADH and cofactor. Activity was monitored via fluorescence increase and additionally observed by eye under a UV-lamp.

Table S5: Typical set-up of ADH assays in microtiter plates.

compound	stock solution		Σ 100 µL	final concentration
Substrate **4** in buffer	8 mM	10×	3.75 µL	300 µM
Polymyxin B in buffer	2 mM	10×	5 µL	100 µM
$NADP^+$ in buffer	12 mM	10×	5 µL	600 µM
Enzyme/cell suspension [1]	-	-	50 µL	-
Buffer (100 mM, KPi, pH 8.0)	-	-	To 100 µL	-

Assay protocol: The reaction was set up in a multi-well plate and incubated at room temperature in the dark overnight; fluorescence was measured at 450/515 nm. A calibration curve was obtained by addition of pyranine 1; different extents of conversions to product were simulated using a dilution series of pyranine.

[1] Cells from 500 µL expression culture (expression induced by addition of anhydrotetracycline) were pelleted by centrifugation. Cell pellets were resuspended in 150 µL buffer (100 mM potassium phosphate, pH 8.0) and the suspension was added to the microtiter plate for lysis and reaction.

10. Primary data for the calculation of enrichment values

Enrichment values were determined quantified by analysis of re-screening experiments of number of recovered clones, as described in the preceding section 9 (see Figure 4 for the workflow). Positive and negative control

**Table S6**	Dilution 1:1000	
	before droplet sorting	*after droplet sorting*
Number of positives	1	15
Number of negatives	999	17
Total number tested	1000	32
Positive fraction	0.001	0.469
Negative fraction	0.999	0.531
Ratio of positives *vs* negatives	0.001001	0.88
Enrichment calculated according to Zinchenko *et al.*[8]	469-fold	
Enrichment calculated according to Baret *et al.* [9]	882-fold	

**Table S7**	Dilution 1:100	
	before droplet sorting	*after droplet sorting*
Number of positives	1	79
Number of negatives	99	9
Total number tested	100	88
Positive fraction	0.01	0.898
Negative fraction	0.99	0.102
Ratio of positives *vs* negatives	0.010101	8.78
Enrichment calculated according to Zinchenko *et al.*[8]	90-fold	
Enrichment calculated according to Baret *et al.* [9]	869-fold	

11. NMR spectra

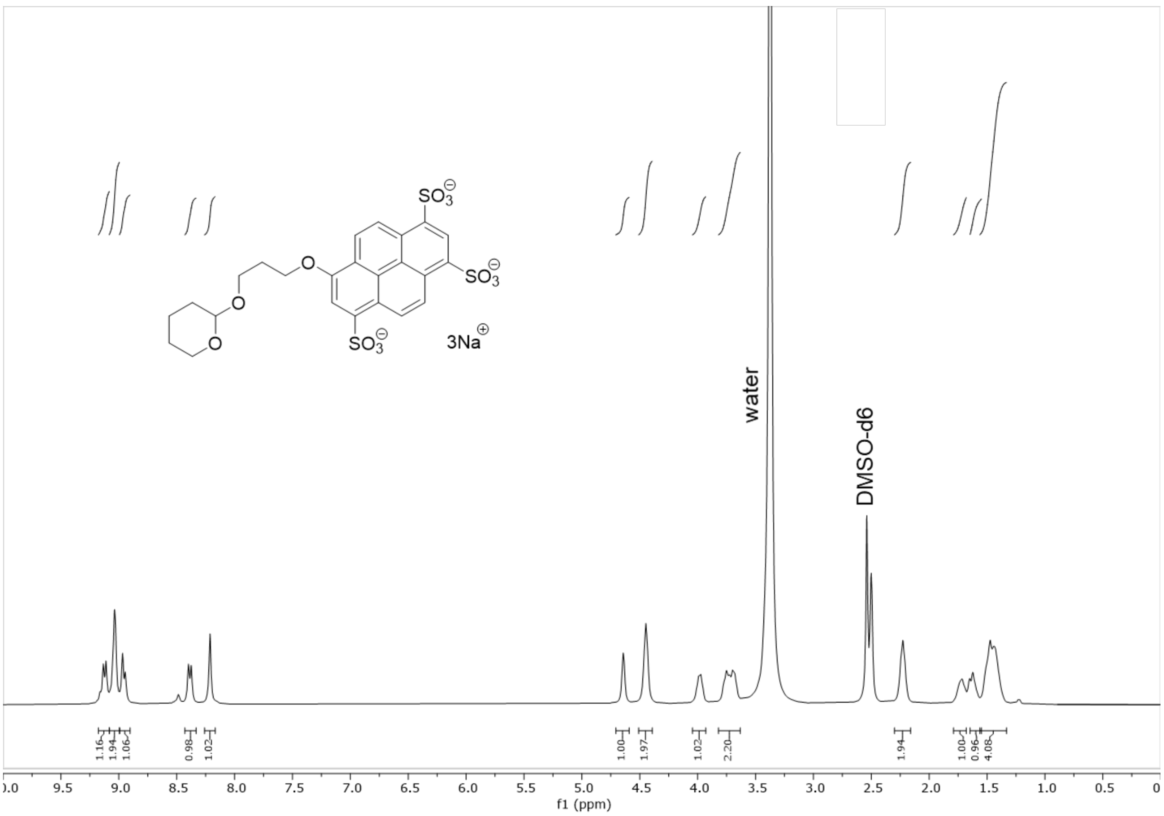

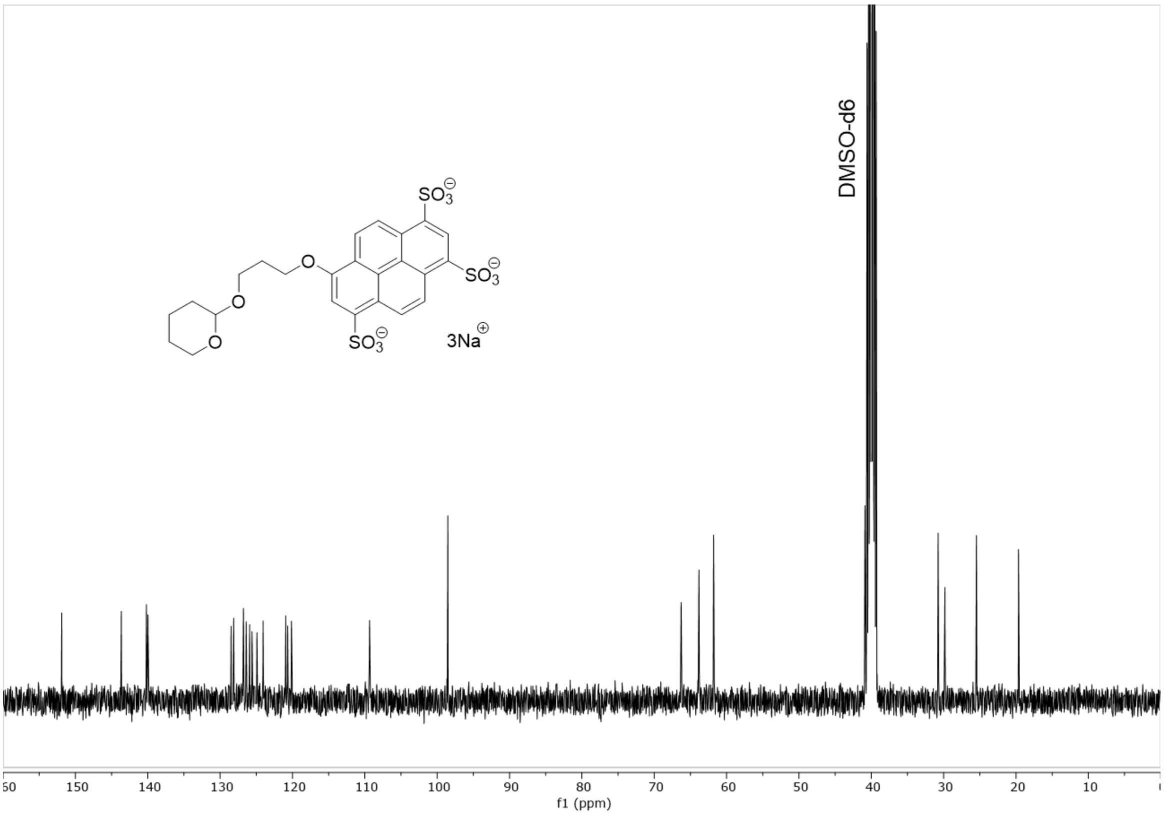

\

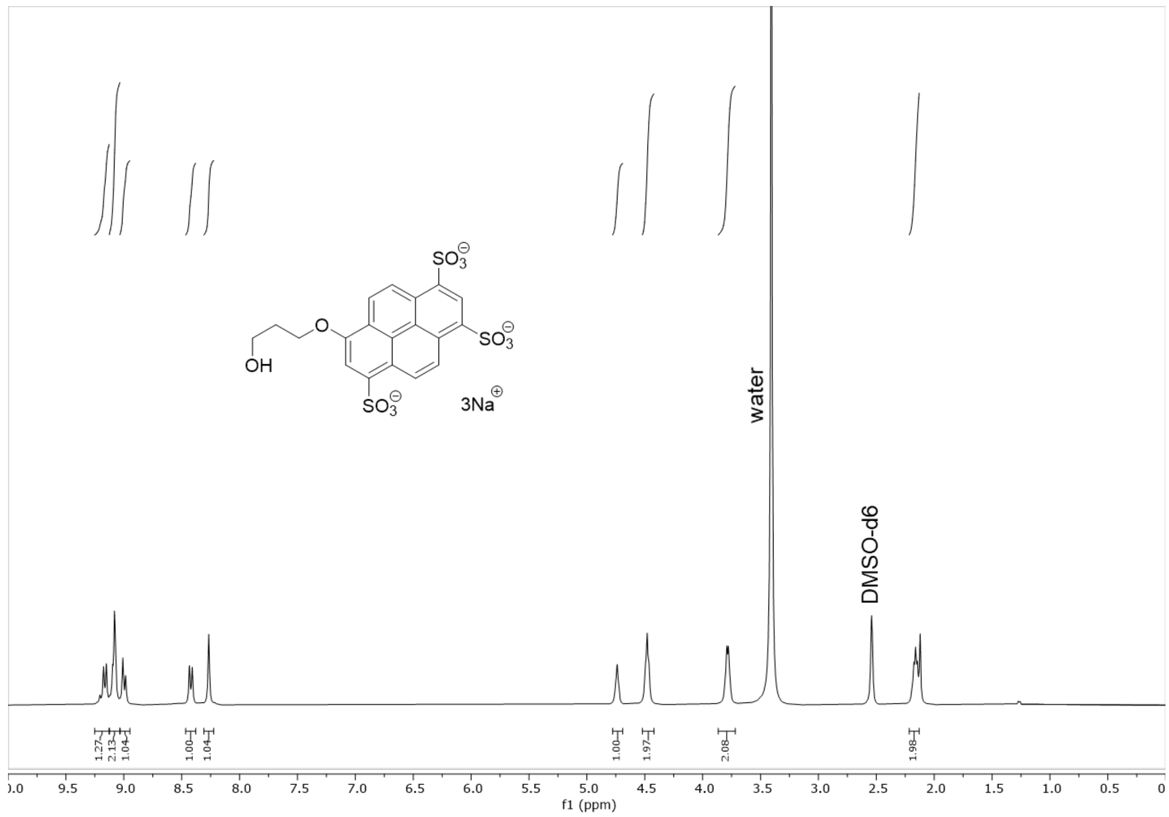
water
DMSO-d6
OH
O
SO3
3Na
f1 (ppm)

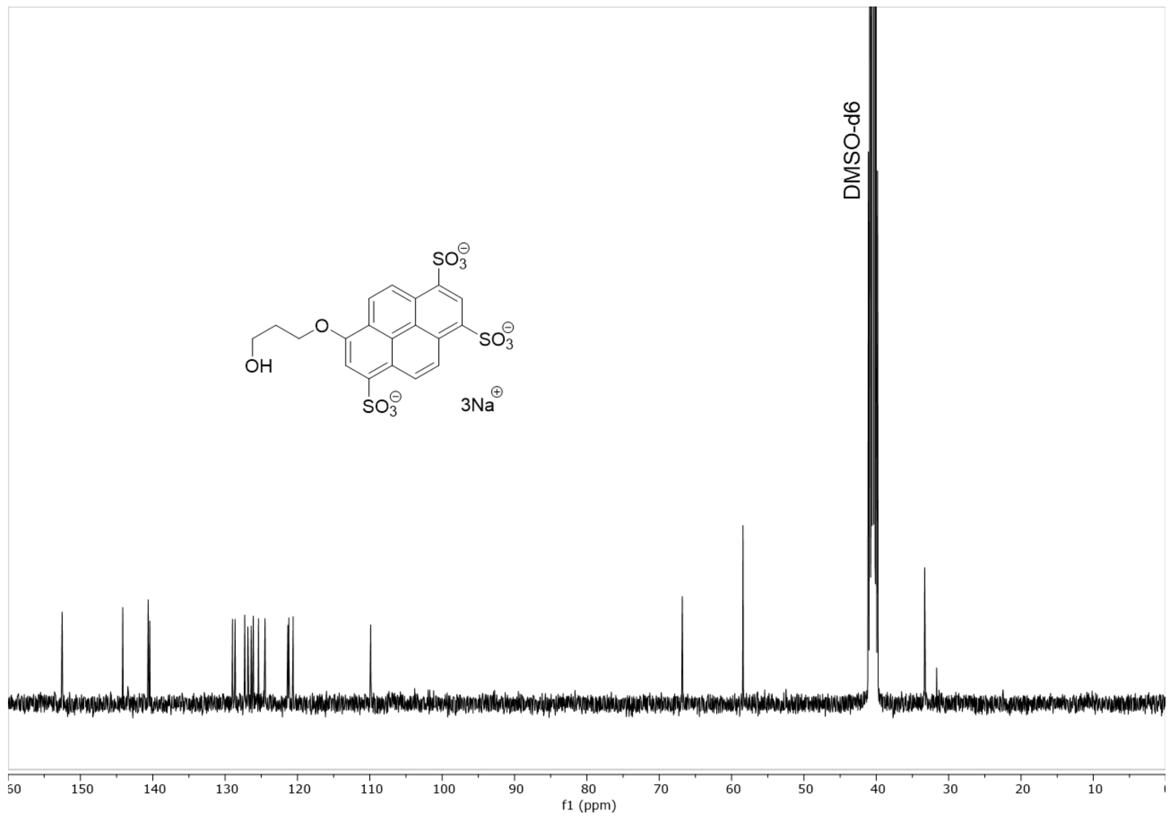
DMSO-d6
OH
O
SO3
3Na
f1 (ppm)

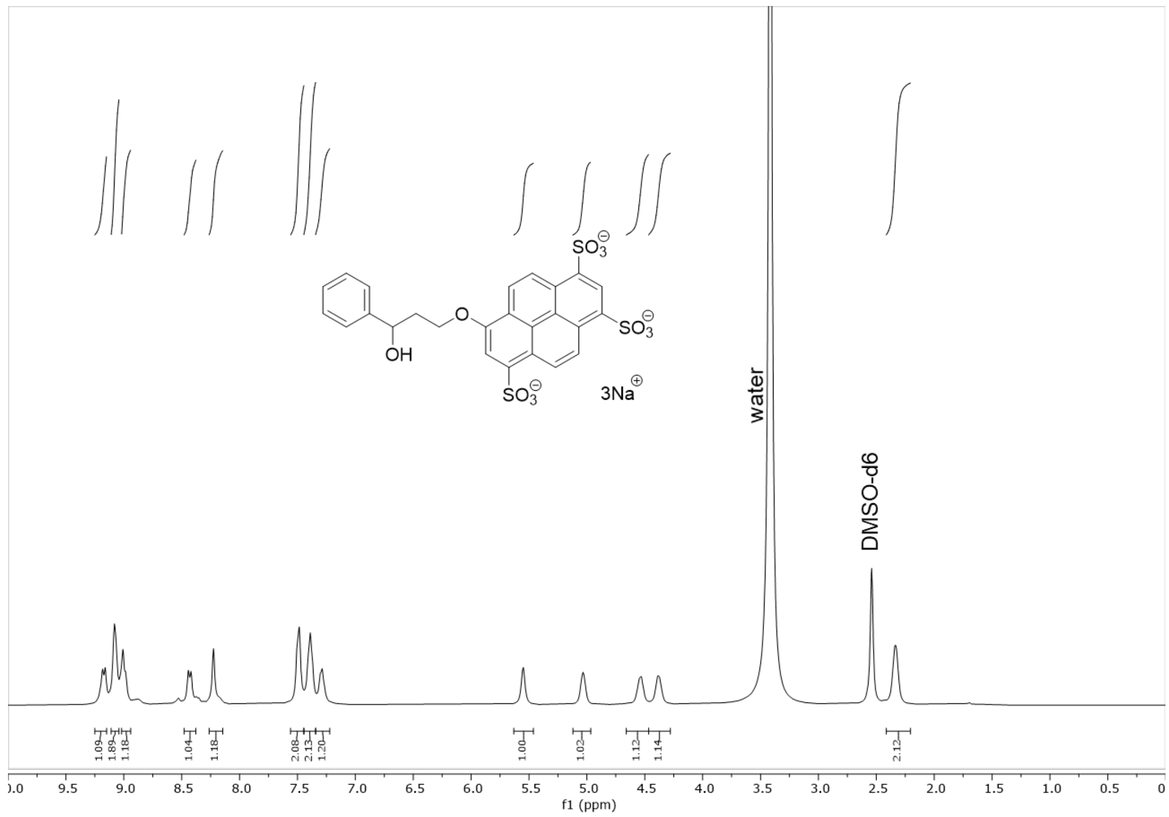
SO3⊖
SO3⊖
OH
O
SO3⊖
3Na⊕
water
DMSO-d6
1.09
1.89
1.18
1.04
1.18
2.08
2.13
1.20
1.00
1.02
1.12
1.14
2.12
f1 (ppm)

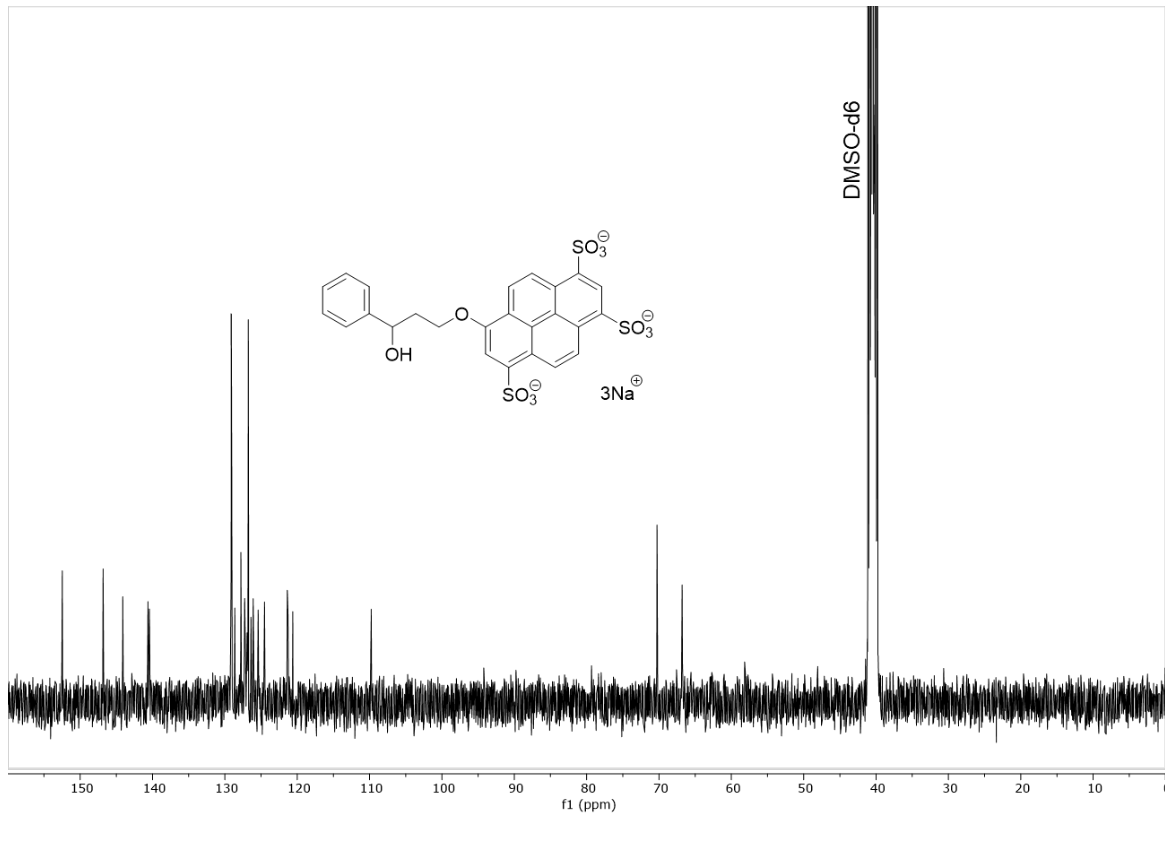
DMSO-d6
SO3⊖
SO3⊖
OH
O
SO3⊖
3Na⊕
f1 (ppm)

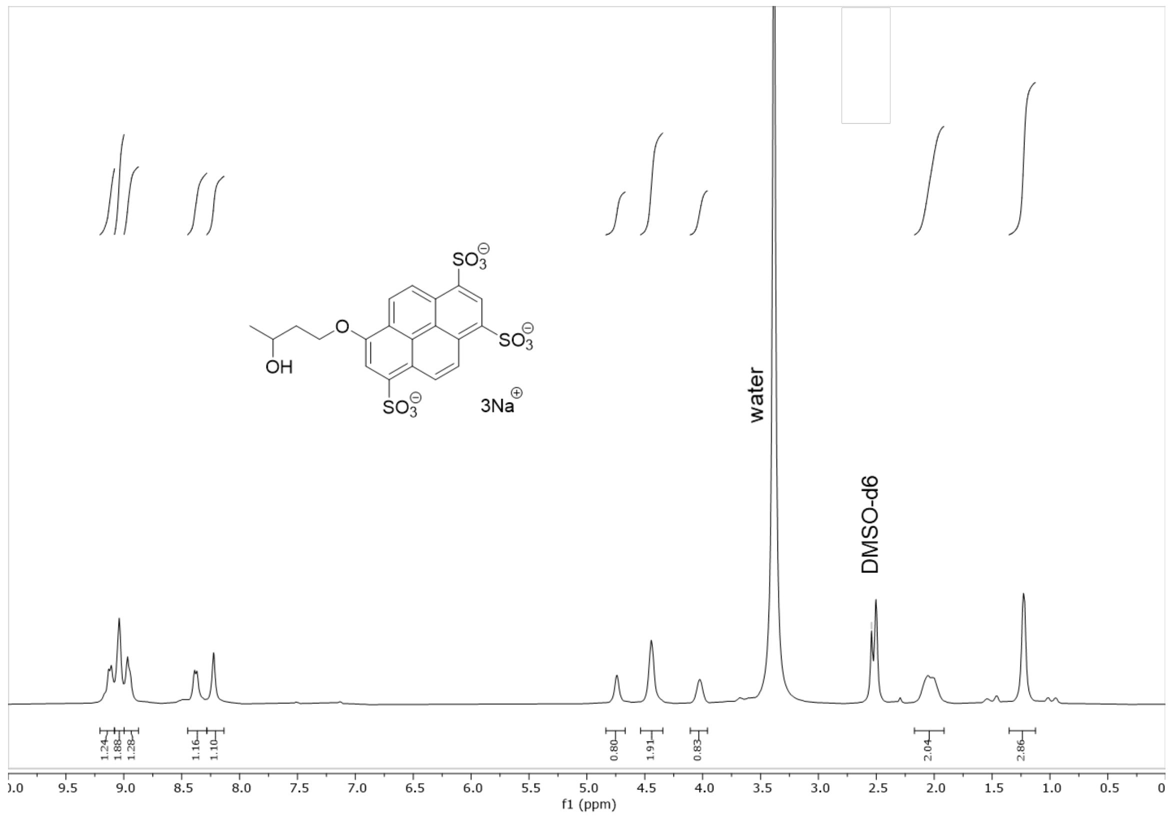

SO3
SO3
OH
SO3
3Na
water
DMSO-d6
1.24
1.88
1.28
1.16
1.10
0.80
1.91
0.83
2.04
2.86
9.5
9.0
8.5
8.0
7.5
7.0
6.5
6.0
5.5
5.0
4.5
4.0
3.5
3.0
2.5
2.0
1.5
1.0
0.5
f1 (ppm)

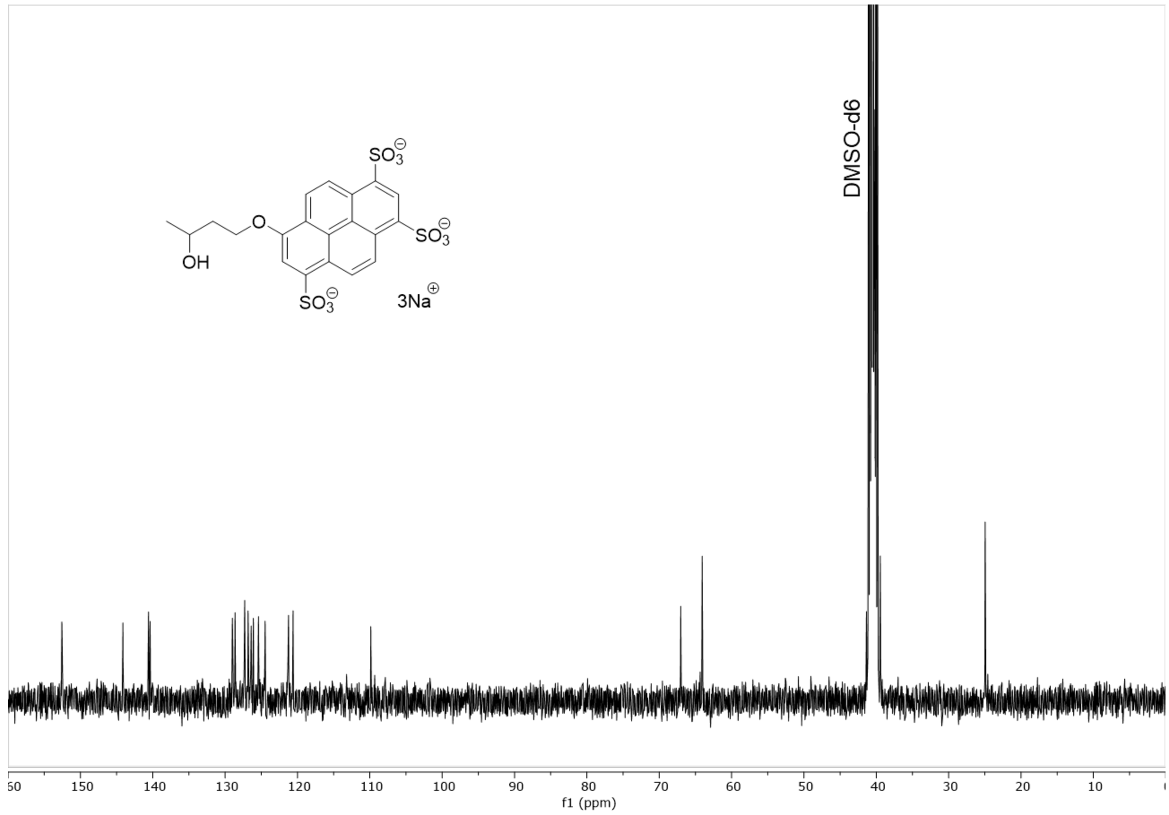

DMSO-d6
SO3
SO3
OH
SO3
3Na
150
140
130
120
110
100
90
80
70
60
50
40
30
20
10
f1 (ppm)

12. Supplementary References

[1] A. Sciambi, A. R. Abate, *Lab Chip* **2014**, *14*, 2605-2609.

[2] F. Gielen, R. Hours, S. Emond, M. Fischlechner, U. Schell, F. Hollfelder, *Proc Natl Acad Sci U S A* **2016**, *113*, E7383-E7389.

[3] H. Goto, Y. Kanai, A. Yotsui, S. Shimokihara, S. Shitara, R. Oyobiki, K. Fujiwara, T. Watanabe, Y. Einaga, Y. Matsumoto, N. Miki, N. Doi, *Lab Chip* **2020**, *20*, 852-861.

[4] D. A. Holland-Moritz, M. K. Wismer, B. F. Mann, I. Farasat, P. Devine, E. D. Guetschow, I. Mangion, C. J. Welch, J. C. Moore, S. Sun, R. T. Kennedy, *Angew Chem Int Ed Engl* **2020**, *59*, 4470-4477.

[5] M. Naumann, J. S. Wooten, **2013**, Patent "Superabsorbent Copolymer", WO 2013/139673 Al

[6] D. Qin, Y. Xia, G. M. Whitesides, *Nat Protoc* **2010**, *5*, 491-502.

[7] (a) B. van Loo, M. Heberlein, P. Mair, A. Zinchenko, J. Schuurmann, B. D. G. Eenink, J. M. Holstein, C. Dilkaute, J. Jose, F. Hollfelder, E. Bornberg-Bauer, *ACS Synth Biol* **2019**, *8*, 2690-2700; (b) J. M. Holstein, C. Gylstorff, F. Hollfelder, *ACS Synth Biol* **2021**, *10*, 252-257.

[8] A. Zinchenko, S. R. Devenish, B. Kintses, P. Y. Colin, M. Fischlechner, F. Hollfelder, *Anal Chem* **2014**, *86*, 2526-2533.

[9] J. C. Baret, O. J. Miller, V. Taly, M. Ryckelynck, A. El-Harrak, L. Frenz, C. Rick, M. L. Samuels, J. B. Hutchison, J. J. Agresti, D. R. Link, D. A. Weitz, A. D. Griffiths, *Lab Chip* **2009**, *9*, 1850-1858.
